# Impact of steroid-sparing immunosuppressive agents on tumor outcome in the context of cancer immunotherapy with highlight on melanoma: a systematic literature review and meta-analysis

**DOI:** 10.3389/fimmu.2024.1499478

**Published:** 2024-12-16

**Authors:** Jennifer Strouse, Karmela Kimi Chan, Rachel Baccile, Gong He, Diana K. N. Louden, Mihai Giurcanu, Arohi Singh, John Rieth, Noha Abdel-Wahab, Tamiko R. Katsumoto, Namrata Singh, Sherin Rouhani, Pankti Reid

**Affiliations:** ^1^ Division of Immunology, University of Iowa, Iowa City, IA, United States; ^2^ Division of Rheumatology, Department of Medicine, Hospital for Special Surgery, New York, NY, United States; ^3^ Center for Health and The Social Sciences, University of Chicago, Chicago, IL, United States; ^4^ Division of Hematology and Oncology, University of Michigan, Ann Arbor, MI, United States; ^5^ University Libraries, University of Washington, Seattle, WA, United States; ^6^ Department of Public Health Sciences, University of Chicago, Chicago, IL, United States; ^7^ University of Chicago Medicine, Chicago, IL, United States; ^8^ Division of Hematology, Oncology, and Blood & Marrow Transplantation, University of Iowa Health Care, Iowa City, IA, United States; ^9^ Section of Rheumatology & Clinical Immunology, Department of General Internal Medicine, and Department of Melanoma Medical Oncology, University of Texas MD Anderson Cancer Center, Houston, TX, United States; ^10^ Department of Rheumatology & Rehabilitation, Assiut University Hospitals, Assiut University Faculty of Medicine, Asyut, Egypt; ^11^ Division of Immunology and Rheumatology, Department of Medicine, Stanford University, Stanford, CA, United States; ^12^ Division of Rheumatology, University of Washington, Seattle, WA, United States; ^13^ Mass General Cancer Center, Massachusetts General Hospital, Boston, MA, United States; ^14^ Division of Rheumatology, Department of Medicine, University of Chicago, Chicago, IL, United States

**Keywords:** cancer immunotherapy, tumor outcome, steroid-sparing agents, disease-modifying antirheumatic drugs (DMARDs), immune related adverse events (irAEs), ICI toxicity, TNF inhibitors (TNFi), biologics

## Abstract

**Background:**

The impact of steroid-sparing immunosuppressive agents (SSIAs) for immune-related adverse events (irAEs) on tumor outcome is not well-known. This systematic review evaluates tumor outcomes for corticosteroid (CS) monotherapy versus CS with SSIA (CS-SSIA) for irAE treatment with a focus on melanoma.

**Methods:**

Search was conducted through 1/5/23 using PubMed, Embase, Cochrane CENTRAL, and Web of Science. We included case series, retrospective/prospective observational studies and interventional clinical trials. Individual-level data was analyzed using KM curves and Cox regression for overall survival (OS) and progression free survival (PFS). Time to SSIA was treated as a time-varying exposure using landmark analysis (landmark timepoint=3 months after irAE) to account for immortal time bias. For group-level data, meta-analysis compared the use of SSIA to No SSIA for irAEs.

**Results:**

Of twenty-two publications with individual-level data, 147 patients with any cancer (57 CS, 90 CS-SSIA) and 65 with melanoma (18 CS, 47 CS-SSIA) underwent landmark analysis. Twenty-two publications underwent group-level evaluation and four were included in the meta-analysis. CS-SSIA versus CS showed higher risk of all-cause mortality and progression (HR 2.75, 95%CI: 1.44-5.27, p<0.01 and HR 1.75, 95%CI: 1.07-2.85, p=0.03, respectively). Melanoma showed worse OS and PFS for CS-SSIA versus CS (HR 5.68, 95%CI: 1.31-24.67, p=0.02 and HR 2.68, 95%CI: 1.12-6.40, p=0.03, respectively). In the meta-analysis of group-level data (n=2558), we found worse OS and PFS for CS-SSIA versus No SSIA (HR 1.58, 95%CI: 1.25; 2.01, p<0.01 and 1.70, 95%CI: 1.25-2.33, p<0.01). Tumor necrosis factor-alpha inhibitors (TNFi) were the most common SSIA. In the melanoma cohort, TNFi had worse OS and PFS versus CS (HR 6.46, 95%CI: 1.43-29.19, p = 0.02 and HR 7.49, 95%CI: 2.29-24.48, p<0.01, respectively). TNFi versus Other SSIAs showed a trend toward worse OS and worse PFS (HR 6.96, 95%CI: 0.90-53.65, p=0.06 and HR 21.5, 95%CI: 2.63-175.8, p<0.01, respectively). Meta-analysis showed a concern for TNFi compared to Other SSIA (HR 1.56, 95%CI: 1.17-2.09, p<0.01 respectively).

**Conclusions:**

While our results raise concern about the effects of CS-SSIA and TNFi for irAE therapy on tumor outcomes, prospective randomized controlled trials are needed to definitively assess the effect of SSIAs on tumor outcomes.

## Introduction

Since initial approval in 2011, immune checkpoint inhibitors (ICIs) have reformed cancer treatment and have improved outcomes in several malignancies. It is estimated that the proportion of patients with cancer eligible to receive one or more of the programmed cell death-1 (PD-1), programmed cell death-ligand 1 (PD-L1), or cytotoxic T lymphocyte associated antigen-4 (CTLA-4) ICIs has increased from 1.5% in 2011 to 43.6% in 2018 (1). As the population of patients eligible for these therapies has expanded, so has the number of patients experiencing immune related adverse events (irAEs).

IrAEs are driven by various mechanisms, including non-specific immune activation, loss of immune tolerance to native host tissues, and amplification of pre-existing autoimmunity (2). IrAEs are heterogenous and unpredictable in their clinical presentation, with variable organ involvement and severity. According to the current practice management guidelines, corticosteroids (CS) remain the mainstay of first-line treatment for most irAEs (3–6). However, high doses and prolonged courses of CS can result in significant toxicities, including the development of opportunistic infections, steroid-induced diabetes, weight gain, and cardiac toxicity (7–9). In severe irAE cases, toxicity may force patients to discontinue an otherwise effective ICI. Additionally, although retrospective reports have noted better tumor outcomes for patients who experience irAEs (10, 11), the use of nonspecific immunosuppression by systemic steroids raises concerns for mitigation of ICI efficacy leading to a negative impact on tumor outcomes (7, 12–14).

As backbone of therapy for most irAEs, systemic steroids are frequently prescribed for irAEs. Yet, CS treatment alone is insufficient in some cases. It is estimated that 11% of patients have steroid-refractory or steroid-dependent irAEs (15–18). For patients who require alternative immunosuppression for irAE management, there is a paucity of evidence regarding which steroid-sparing immunosuppressive agents (SSIAs) are most effective at treating the irAE and have the lowest risk of reducing the effectiveness of immunotherapy.

Concerns have been raised that some agents, including TNF-alpha inhibitors (TNFi), may impair the anti-tumor immune response (19, 20); however, investigations in melanoma murine models suggest TNF inhibition may actually augment anti-tumor immunity (21, 22). A translational study evaluating colonic biopsies from patients with melanoma and ICI-colitis suggested that IL-6 blockade may not compromise ICI efficacy, which was further validated in a murine model (23). Until adequately-powered prospective randomized controlled clinical trials are published, providers rely on currently available guidance based on observational studies and expert opinion.

The primary aim of this systematic literature review was to determine the effect of SSIAs for irAE treatment on tumor outcomes with a focus on melanoma. We also analyzed the impact on tumor outcomes for TNFi versus CS and for TNFi compared to other SSIAs used for irAE management. We contextualize the most up-to-date evidence regarding TNFi based on current literature and underscore the need to further investigate other SSIAs, particularly IL-6 axis antagonists. We also highlight the missingness that exists in published data within this area of research and provide guidance for future research.

## Methods

The current review follows the methods outlined in an established protocol registered with PROSPERO (CRD42021292867). It is reported in accordance with PRISMA guidelines.

### Search strategy and study selection

With input from the clinical investigators, a systematic search was conducted by a research librarian in four databases: PubMed, Embase, Cochrane Central Register of Controlled Trials, and the Web of Science Core Collection. The search strategies (**Supplementary 1** in **Supplementary Data Sheet 1**) incorporated text words and controlled vocabulary terms appropriate to each database to represent the following three concepts: ICIs, SSIA(s), and clinical studies or case series. The literature search was initially conducted on December 10, 2021, and was updated on January 5, 2023. Search results were limited to English-language journal articles and registered trials, and no date limits were applied.

We included case series, retrospective and prospective observational studies and interventional clinical trials published as manuscripts. Inclusion criteria: patients ≥18 years old with solid tumor treated with ICI(s) and >=1 SSIA(s) with tumor outcomes, overall survival (OS) and/or progression free survival (PFS) data, reported after at least 3 months of follow-up. This manuscript specifically focuses on studies where SSIAs were used for irAE management, and we did not include patients who used SSIAs for pre-existing autoimmune diseases or transplant therapy or concurrent start of SSIA with ICI(s) for the goal of preventing ICI toxicities. To reduce selection bias and publication bias, we only included case series where all patients with the condition of interest during the given dates were included; case studies and case series where only a select few representative patients were discussed were excluded. The CS only cohort was derived from any patients treated with corticosteroids only included in papers that also included patients treated with SSIA(s); papers that only included a CS cohort and did not include an SSIA cohort were not included.

We then divided the publications into two different categories: (1) studies that had detailed enough extractable information on patients and their tumor outcomes, which we grouped into “Individual-level data” analysis and (2) studies that did not have granular enough information for individual patient-level data but did have information for a group of patients: we denoted as “Group-level data” analysis.

### Data extraction

Data collection was performed independently by seven authors (JS, KKC, GH, TRK, NS, SR, PR) and discrepancies were resolved by discussion. Data extraction was conducted differently for studies with granular enough information to gather individual-level data compared with studies that only reported enough information to conduct group-level data analysis. One study had enough information for both individual-level data but also group-level data: Lesage, et al., *J Immunother*, 2019.

#### Individual-level data extraction

For the publications with available individual-level data reported for analysis (**Supplementary 2A** in **Supplementary Data Sheet 1**), the following information was extracted: (1) study characteristics: study design, journal title, year of publication; (2) patient characteristics: age, sex; (3) cancer diagnosis and treatment: tumor type, stage, number of prior treatments, ICI regimen used, ICI duration, additional therapy (chemotherapy, radiotherapy, tyrosine-kinase inhibitor); (4) irAE details: primary irAE for which immunosuppression was used, time of irAE onset from ICI initiation (months), irAE severity as measured by Common Terminology Criteria for Adverse Events (CTCAE) (5) irAE treatment(s) for CS +/- SSIA(s): timing of treatment start, duration of therapy, and for those with SSIA treatment: number of SSIA(s) if more than one, SSIA regimen (conventional, biologic, targeted synthetic); (6) study endpoints: OS and PFS; and overall response rate (ORR); irAE outcome and (5) toxicity: ICI discontinuation, irAE treatment adverse events, infection.

#### Group-level data extraction

Group-level data was abstracted for publications without sufficient granularity to collect individual-level information (**Supplementary 2B** in **Supplementary Data Sheet 1**) and the following information was collected: (1) study characteristics: study design, journal title, year of publication; (2) patient characteristics: total number of patients in each cohort, patient demographics if available; (3) cancer diagnosis and treatment: tumor type, stage, number of prior treatments, ICI regimen used, ICI duration; (4) irAE details: primary irAE for which SSIA was used, time of irAE onset form ICI initiation (months), irAE severity as measured by CTCAE rubric (5) irAE treatment(s) for CS +/- SSIA(s): timing of treatment start, duration of therapy, and for those with SSIA treatment: number of SSIA(s) if more than one, SSIA regimen (conventional, biologic, targeted synthetic); (6) study endpoints: adjusted Hazard Ratio (aHR) for OS and PFS, other PFS details, OS details, objective response rate (ORR).

### Statistical analysis

Statistical analysis was performed in an iterative fashion and separately for data that had sufficient individual-level details available versus data that required group-level analysis.

Comparator groups were as follows:

- For individual-level data, patients treated with SSIAs in addition to CS (CS-SSIA) were compared to patients treated with CS monotherapy (CS).
- For group-level data, it was not consistently clear whether the entire control group was treated with systemic corticosteroids, so we compared two cohorts: SSIA versus No SSIA (no use of any SSIAs for irAE).

#### Individual-level data statistical analysis

Patients in CS cohort were compared to those in CS-SSIA cohort on several baseline characteristics to assess balance and data missingness patterns using chi-squared tests for categorical data and t-tests for continuous data.

We then conducted landmark analyses comparing OS and PFS for the CS-SSIA vs the CS cohorts. Since SSIA is generally a second line therapy after CS, we considered the time-dependent exposure of the SSIA cohort using landmark analysis (24, 25). The landmark time point was set as 3 months after irAE onset; thus, any subject who was lost to follow up or experienced an outcome event (death of any cause/progression) prior to this time was excluded from the data set, and subjects who had SSIA by the landmark time were classified as CS-SSIA, and all patients who either had CS monotherapy or had SSIA after the landmark time were classified as CS.

Additional statistical assumptions for OS and PFS were considered. For OS, if OS time was missing and PFS time was not missing and OS status was 0 (alive), then OS time = PFS time and if either OS time was missing or OS status was missing and irAE time was not missing, then OS time = irAE time and OS status was set to 0 (alive). For PFS, if either PFS time or PFS status was missing and OS time was not missing and OS status was 1 (death), then PFS time = OS time and PFS status = 1 (progression or death). Further search in the corresponding publications revealed that patients who had PFS time missing but had OS time nonmissing and OS status was death, they did not have progression before death. After these additional checks and edits, there were no missing data for OS data while for PFS, data was available for n=49 CS patients and n=148 CS-SSIA patients.

Our primary outcomes were OS and PFS. We calculated Kaplan-Meier curves and conducted Cox regression analyses. We also conducted subgroup analyses comparing CS use versus CS plus TNFi (TNFi), and CS plus TNFi use (TNFi) versus CS plus other SSIAs (Other SSIA) among all tumor types and for the melanoma subgroup. P-values of <0.05 were considered significant. All analyses were done using statistical software R (26), version 4.2.2, survival analysis using the R package survival, version 3.5-8 (27), and Kaplan-Meier curves using the R package ggsurvfit, version 1.0.0 (28).

#### Group-level data statistical analysis

Summary statistics were extracted from studies that only reported group-level tumor outcomes without providing individual-level detail. For those studies that presented their group-level outcomes in figures, we calculated estimates via extraction of curve data. However, for absolute accuracy and to avoid inherent errors in calculating estimations from figures, we only included studies with provided adjusted hazard ratio (aHR) in the meta-analyses. We used the fixed effects model for aHRs (with corresponding 95%CI reported from the publications). We calculated the pooled estimates of aHR using the inverse variance method and the test of heterogeneity between the studies was calculated using the DerSimonian and Laird method (29). The meta-analysis was conducted using the R package meta (30, 31).

### Quality assessment

Two authors (JR and AS) independently assessed the quality of each publication as poor, fair, or good. Studies with a rating of good had the least risk of bias. Differences were resolved by discussion between the first and second reviewers. Study quality of cohort studies was assessed using the Newcastle-Ottawa scale (NOS). Briefly, the NOS was developed to assess the quality of nonrandomized studies with its design, content and ease of use directed to the task of incorporating the quality assessments in the interpretation of meta-analytic results. Each study was evaluated based on three broad perspectives: the selection of the study groups; the comparability of the groups; and the ascertainment of the outcome of interest. Study quality of case series studies was assessed using the NIH Quality Assessment Tool for Case Series Studies scale. Each study was judged on the clarity of the study’s objective, the clarity of the study population, consecutiveness of cases, comparability of subjects, length of follow-up, and clarity of interventions, outcomes, and statistical methods. Each case series was then given a quality rating of good, fair, or poor (**Supplementary 3** in **Supplementary Data Sheet 1**).

## Results

### Identification of eligible studies

**Figure 1:**
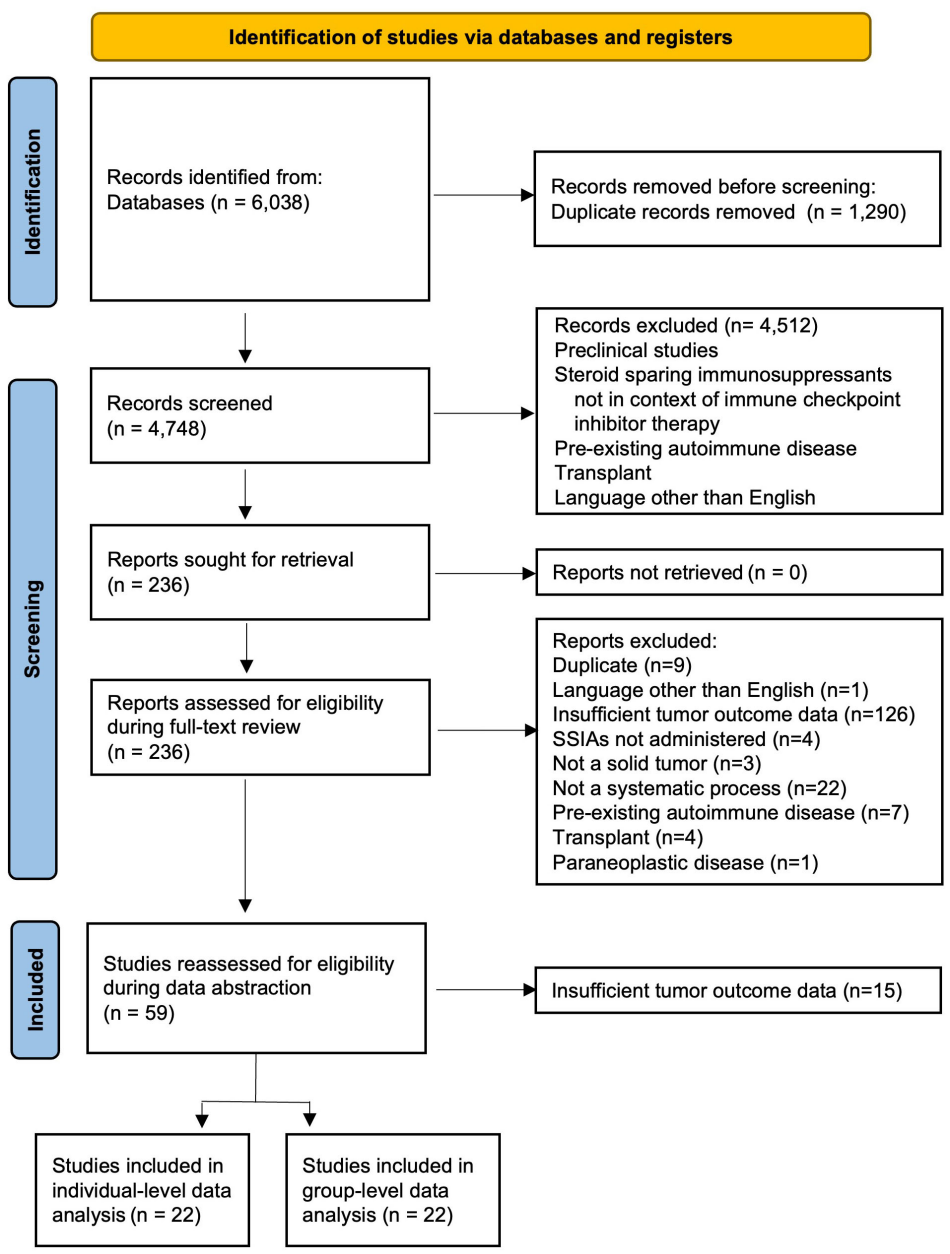
PRISMA flow diagram.

A total of 6038 references were retrieved (**Figure 1**). After 1290 duplicates were removed using Covidence (32), 4748 title and abstracts were screened and 236 were assessed for eligibility and underwent full-text review. The most common reason for exclusion after full text review was lack of sufficient information for tumor outcome and therefore the inability for this data to contribute to the primary outcome of our review and meta-analysis. Ultimately, data was abstracted from 59 studies and after this data-abstraction, it was deemed that 22 studies (33–54) had sufficient information for group-level data analysis and 22 studies (17, 19, 20, 42, 55–71) had sufficient information for more in-depth individual-level data analysis.

### Study and patient characteristics

Data was abstracted from 44 studies, of which 22 had individual-level data and 22 had group-level data. Study characteristics are described in **Table 1**.

**Table 1:** Study characteristics.

INDIVIDUAL-LEVEL DATA MANUSCRIPTS									
Authors	Type of study	Total n	Comparator arm within manuscript	Malignancy type	Primary irAE for which SSIA used	SSIA details	n for SSIA arm	Comparator arm 1	n for comparator arm 1
Camard, et al., *Resper Med and Res*, 2022 (33)	Cohort Study	6	NA	Mix	Pneumonitis	Mix of SSIAs	6	NA	0
Campochiaro, et al., *Eur J Intern Med*, 2021 (34)	Case Series	5	NA	Mix	Mixed	Mix of SSIAs	5	NA	0
Cortazar, et al., *JASN*, 2020 (35)	Cohort Study	8	NA	Mix	Nephritis	Mix of SSIAs	8	NA	0
De La Fuente, et al., *RMD Open*, 2022 (36)	Cohort Study	19	NA	Mix	Arthritis	Mix of SSIAs	19	NA	0
Galmiche, et al., *J Eur Acad Dermatol Venereol*, 2019 (37)	Cross-sectional study	5	Yes, Steroid monotherapy	Melanoma	Encephalitis	IVIg	2	Steroid monotherapy	3
Izumi, et al., *Mol Clin Oncol*, 2019 (38)	Case Series	2	Yes, Steroid monotherapy	NSCLC	Mixed	Mycophenolate	1	Steroid monotherapy	1
Johncilla, et al., *Histopathology*, 2020 (39)	Cross-sectional study	12	Yes, Steroid monotherapy	Mix	Gastritis	TNFi (Infliximab)	2	Steroid monotherapy	10
Kim, et al., *Ophthalmology*, 2019 (40)	Case Series	12	Yes, Steroid monotherapy	Melanoma	Mixed	Mix of SSIAs	3	Steroid monotherapy	9
Larkin, et al., *Oncologist*, 2017 (41)	Case Series	4	Yes, Steroid monotherapy	Melanoma	Encephalitis	IVIg	3	Steroid monotherapy	1
Lesage et al., *J Immunother*, 2019 (42)	Cohort Study	27	NA	Melanoma	Colitis	TNFi (Infliximab)	27	NA	0
Lidar et al., *Autoimmun Rev*, 2018 (43)	Cross-sectional study	14	Steroid monotherapy	Melanoma	Mixed	Methotrexate	8	Steroid monotherapy	6
Lin, et al., *Oncoimmunology*, 2021 (44)	Cross-sectional study	10	NA	Mixed	Nephritis	TNFi (Infliximab)	10	NA	0
Luo, et al., *J Clin Oncol, 2021* (45)	Cohort Study	44	NA	NSCLC	Mixed	Mix of SSIAs	44	NA	0
Mamlouk, et al., *J Immunother Cancer*, 2020 (46)	Cross-sectional study	5	NA	Mix	Vasculitis	Rituximab	5	NA	0
Mitchell, et al., *Eur J cancer*, 2018 (47)	Case Series	18	Yes, Steroid monotherapy	Mix	Mixed	Mix of SSIAs	8	Steroid monotherapy	10
Miyahara, et al., *Digestion*, 2020 (48)	Case Series	2	Yes, Steroid monotherapy	NSCLC	Colitis	TNFi (Infliximab)	1	Steroid monotherapy	1
Mohn, et al., *Melanoma Research*, 2019	Case Series	4	Yes, Steroid monotherapy	Melanoma	Mixed	IVIg	3	Steroid monotherapy	1
Nakagomi, et al., *Front Pharmacol*, 2022 (50)	Case Series	4	Yes, Steroid monotherapy	Mix	Myositis/Myocarditis	IVIg	2	Steroid monotherapy	2
O’Reilly, et al., *Support Care Cancer*, 2020 (51)	Cohort Study	3	Yes, Steroid monotherapy	Melanoma	Hepatitis	Methotrexate	1	Steroid monotherapy	2
Sagiv, et al., *JAMA Ophthalmol*, 2018 (52)	Case Series	2	Yes, Steroid monotherapy	Melanoma	Colitis	TNFi (Infliximab)	1	Steroid monotherapy	1
Segui, et al., *Ann Gastroenterol*, 2022	Case Series	20	Yes, Steroid monotherapy	Mix	Mixed	Mix of SSIAs	6	Steroid monotherapy	14
Taliansky, et al., *Support Care Cancer*, 2021 (54)	Cross-sectional study	5	Yes, Steroid monotherapy	Mix	Mixed	Cyclophosphamide	1	Steroid monotherapy	4

GROUP-LEVEL DATA MANUSCRIPTS									
Authors	Type of study	Total n	Comparator arm within manuscript	Malignancy type	Primary irAE for which SSIA used	SSIA details	n for SSIA arm	Comparator arm 1	n for comparator arm 1
Abu-Sbeih, et al., *J Immunother Cancer*, 2019 (55)	Cohort study	84	SSIA vs. no SSIA	Mix	Colitis	TNFi (Infliximab +/- Vedo vs. Vedo in KM)	52	No systemic, but yes Vedolizumab	32
Alexander, et al., *J Immunother Cancer*, 2021 (56)	Cohort study	127	NA	Mix	Colitis	TNFi (infliximab) +/- other SSIAs	111	NA	NA
Arriola, et al., *Clin Cancer Res*, 2015 (57)	Cohort study	36	SSIA vs. no SSIA	Melanoma	Mixed	TNFi (Infliximab)	7	Steroid monotherapy	29
Bass, et al., *Ann Rheum Dis*, 2023 (58)	Cohort study	134	NA (SSIA vs another SSIA)	Mix	Arthritis	Mix of SSIAs	134	NA	NA
Burdett, et al., *Asia Pac J Clin Oncol*, 2020 (59)	Case series	19	NA	Mix	Mixed	Mix of SSIAs	19	NA	NA
Chan, et al., *ACR Open Rheumatol*, 2020 (60)	Cohort study	13	NA	Mix	Mixed	Mix of SSIAs (cDMARDs)	13	NA	NA
Cheung, et al., *Frontline Gastroenterol*, 2019 (61)	Cohort study	19	SSIA vs. no SSIA	Mix	Hepatitis	TNFi (Infliximab) + MMF/tacrolimus	8	Steroid monotherapy	11
Cheung, et al., *Br J cancer*, 2020 (62)	Cohort study	134	Infliximab vs no infliximab	Mix	Colitis	TNFi (Infliximab)	29	Steroid monotherapy	105
Dahl, et al., *Aliment Pharmacol Ther*, 2022 (63)	Cohort study	509	SSIA vs. no SSIA	Mix	Colitis	TNFi (Infliximab) +/- vedolizumab	140	No SSIA	369
Dearden, et al., *Eur J Cancer, 2021* (64)	Cohort study	56	SSIA vs. no SSIA	Melanoma	Mixed	Mix of SSIAs	17	Steroid monotherapy	39
Dimitriou, et al., *Cancers (Basel)*, 2021 (65)	Cohort study	108	SSIA vs. no SSIA	Melanoma	Mixed	Mix of SSIAs (Inflix or Toci)	34	Steroid monotherapy	74
Hughes, et al., *Cancer Medicine*, 2019 (17)	Cohort study	60	Any SSIA	Melanoma	Colitis	Mix of SSIAs	30 (28 infliximab)	steroid monotherapy	27
Johnson, et al., *J Immunother Cancer*, 2018 (66)	Cohort Study	40	SSIA vs. no SSIA	Melanoma	Colitis	TNFi (Infliximab)	18	Steroid monotherapy	22
Lesage, et al., *J Immunother Cancer*, 2019 (42)	Cohort study	27	NA	Melanoma	Colitis	TNFi (Any)	27	NA	NA
Mooradian, et al., *J Immunother Cancer*, 2020 (67)	Cohort study	82	SSIA vs. no SSIA	Melanoma	Colitis	TNFi (Infliximab)	35	Steroid monotherapy	47
Nahar, et al., *J Immunother Cancer*, 2020 (68)	Cohort study	100	SSIA vs. no SSIA	Melanoma	Colitis	TNFi (Inflix sensitive, Infliximab refractory)	48	Steroid monotherapy	52
Stroud, et al., *J Clin Oncol*, 2017 (69)	Cohort study	87	SSIA vs. no SSIA	Lung NOS	Mixed	Toci	34	Steroid monotherapy	53
van Not, et al., *JAMA Oncol, 2022* (19)	Cohort study	347	SSIA vs. no SSIA	Melanoma	Mixed	TNFi (Infliximab), Mix of SSIAs	67, 115	Steroid monotherapy	232
Verheijden, et al., *Clin Cancer Res, 2020* (70)	Cohort study	222	SSIA vs no SSIA	Melanoma	Mixed	TNFi (Any)	65	Steroid monotherapy	157
Wang, et al., *J Immunother Cancer*, 2018 (8)	Cohort Study	79	TNFi vs. no SSIA	Mix	Colitis	TNFi (Infliximab)	35	Steroid monotherapy	44
Zhang et al., *JGH Open*, 2021 (71)	Case series	11	NA	Melanoma	Colitis	TNFi (Any)	11	NA	NA
Zou, et al., *J Immunother Cancer*, 2021 (20)	Cohort study	184	SSIA vs. no SSIA	Mix	Colitis	TNFi (Infliximab) +/- Vedo vs. Vedo+Inf	94	No SSIA, vedo without inf	62

#### Individual-level data

Baseline patient characteristics for the 22 publications with enough detail to analyze individual-level data are summarized in **Table 2**. Of the total n=236 patients from these studies, n=174 had received one or more SSIA(s) for irAE treatment and in the CS-SSIA cohort and 62 in CS cohort (**Table 2A**). A total of 115 (53%) patients had melanoma, n=72 (33%) had NSCLC. There was no significant difference in demographics (age, sex) between the two groups. The most common irAE was colitis (29%) with arthritis as the second most common (23%), and colitis was more frequent in the CS-SSIA group compared to CS alone (32.2% vs 19.4%). There was no significant difference in time from ICI initiation to irAE between CS-SSIA and CS groups. There was a substantial amount of missing data – we found that age, sex, ICI duration, irAE duration, and steroid durations were frequently not reported in the primary articles (**Supplementary 4** in **Supplementary Data Sheet 1**). Of the patients with melanoma, n=68 were in CS-SSIA group and n=47 in CS group. The most common SSIA class was TNFi (44% of all patients with any tumor type, 52% of patients with melanoma). Median time from irAE to SSIA initiation for patients with any tumor type was 2.00 months (IQR 0.50, 6.00), for melanoma was 1.78 months (IQR 0.8, 2.5).

**Table 2:** Baseline characteristics for patients with individual-level data.

A. All patients analyzed, not limited to landmark analysis.						
	Any Tumor Type (236)			Melanoma (115)		
	CS	CS-SSIA	p	CS	CS-SSIA	p
n	62	174		47	68	
Demographics						
Age, median [IQR]	67 [53, 72]	64 [55, 71]	0.917	64 [53, 71]	62 [55, 69]	0.863
Age Unknown (%)	12 (19.4)	104 (59.8)	<0.001	10 (21.3)	34 (50.0)	0.003
Sex (%)						
Male	25 (40.3)	37 (21.3)	<0.001	18 (38.3)	15 (22.1)	0.007
Female	25 (40.3)	33 (19.0)		19 (40.4)	19 (27.9)	
Unknown	12 (19.4)	104 (59.8)		10 (21.3)	34 (50.0)	
Cancer Details						
Tumor Type (%)						
Melanoma	47 (75.8)	68 (39.1)	<0.001	47 (100.0)	68 (100.0)	–
NSCLC	6 (9.7)	66 (37.9)		–	–	–
RCC	2 (3.2)	6 (3.4)		–	–	–
GI	2 (3.2)	5 (2.9)		–	–	–
Breast	0 (0.0)	3 (1.7)		–	–	–
Head and Neck	1 (1.6)	2 (1.1)		–	–	–
Other	4 (6.5)	7 (4.0)		–	–	–
Unknown	0 (0.0)	17 (9.8)		–	–	–
ICI Type (%)						
Combination	24 (38.7)	47 (27.0)	0.012	22 (46.8)	25 (36.8)	0.422
Monotherapy	38 (61.3)	108 (62.1)		25 (53.2)	42 (61.8)	
Unknown	0 (0.0)	19 (10.9)		0 (0.0)	1 (1.5)	
ICI Duration, median [IQR]	1.8 [0.8, 7.1]	3.9 [1.5, 11.0]	0.102	2.6 [0.8, 7.8]	3.4 [1.5, 10.5]	0.556
ICI Duration Unknown (%)	30 (48.4)	102 (58.6)	0.213	23 (48.9)	40 (58.8)	0.392
Additional Therapy (%)						
Chemo	1 (1.6)	16 (9.2)	0.101	0 (0.0)	0 (0.0)	0.490
RT	0 (0.0)	8 (4.6)		0 (0.0)	0 (0.0)	
Other	0 (0.0)	1 (0.6)		0 (0.0)	0 (0.0)	
None	26 (41.9)	61 (35.1)		19 (40.4)	22 (32.4)	
Unknown	35 (56.5)	88 (50.6)		28 (59.6)	46 (67.6)	
irAE Details						
Time to irAE, median [IQR]	1.8 [0.7, 7.0]	2.9 [1.0, 6.0]	0.334	2.5 [0.8, 7.9]	2.0 [1.0, 4.0]	0.751
Time to irAE Unknown (%)	21 (33.9)	16 (9.2)	<0.001	14 (29.8)	7 (10.3)	0.016
irAE Duration	3.0 [1.5, 3.5]	3.0 [1.3, 8.0]	0.338	3.0 [2.3, 3.3]	0.8 [0.7, 2.1]	0.567
irAE Duration Missing	55 (88.7)	153 (87.9)	1.000	43 (91.5)	61 (89.7)	1.000
Primary irAE (%)						
Arthritis	14 (22.6)	39 (22.4)	<0.001	11 (23.4)	13 (19.1)	0.016
Colitis	12 (19.4)	56 (32.2)		10 (21.3)	29 (42.6)	
Dermatologic	1 (1.6)	0 (0.0)		0 (0.0)	0 (0.0)	
Encephalitis	8 (12.9)	5 (2.9)		6 (12.8)	5 (7.4)	
Gastritis	7 (11.3)	3 (1.7)		5 (10.6)	2 (2.9)	
Hematologic	0 (0.0)	2 (1.1)		0 (0.0)	0 (0.0)	
Hepatitis	3 (4.8)	7 (4.0)		3 (6.4)	2 (2.9)	
Myositis/Myocarditis	4 (6.5)	9 (5.2)		2 (4.3)	3 (4.4)	
Nephritis	4 (6.5)	19 (10.9)		2 (4.3)	7 (10.3)	
Ocular	3 (4.8)	0 (0.0)		3 (6.4)	0 (0.0)	
Other	2 (3.2)	8 (4.6)		1 (2.1)	2 (2.9)	
Peripheral neuropathy	0 (0.0)	4 (2.3)		0 (0.0)	3 (4.4)	
Pneumonitis	4 (6.5)	14 (8.0)		4 (8.5)	0 (0.0)	
Vasculitis	0 (0.0)	8 (4.6)		0 (0.0)	2 (2.9)	
irAE Treatment Details						
Steroid Dur, median [IQR]	4.0 [3.0, 6.0]	3.0 [1.2, 5.1]	0.301	4.0 [3.0, 6.5]	3.0 [1.2, 5.0]	0.339
Steroid Duration missing	45 (72.6)	106 (60.9)	0.137	34 (72.3)	55 (80.9)	0.395
SSIA Dur, median [IQR]	–	2.0 [0.5, 6.0]	–	–	4.0 [1.0, 6.0]	–
SSIA Duration missing	–	55 (31.6)	–	–	39 (57.4)	–
SSIA Type (%)						
TNFI	–	77 (44.3)	–	–	35 (51.5)	–
Other SSIA	–	69 (39.7)	–	–	21 (30.9)	–
Immunomodulating agent	–	28 (16.1)	–	–	12 (17.6)	–
SSIA Regimen (%)						
Biologic + Conventional	–	20 (11.5)	–	–	1 (1.5)	–
Biologic only	–	95 (54.6)		–	41 (60.3)	
Conventional only	–	27 (15.5)		–	13 (19.1)	
IVIg	–	18 (10.3)		–	9 (13.2)	
Other	–	13 (7.5)		–	3 (4.4)	

B. Patients analyzed in the landmark analysis only.						
	Any Tumor Type (147)			Melanoma (65)		
	CS	CS-SSIA	p	CS	CS-SSIA	p
n	57	90		18	47	
Demographics						
Age, median [IQR]	67 [53, 71]	63 [56, 71]	0.796	66 [53, 68]	62 [55.3, 67.8]	0.832
Age Unknown (%)	29 (50.9)	53 (58.9)	0.434	1 (5.6)	25 (53.2)	0.001
Sex (%)						
Male	12 (42.9)	15 (40.5)	1	5 (29.4)	6 (27.3)	1
Female	16 (57.1)	22 (59.5)		12 (70.6)	16 (72.7)	
Unknown	29 (50.9)	53 (58.9)	0.434	1 (5.6)	25 (53.2)	0.001
Cancer Details						
Tumor Type (%)						
Melanoma	18 (40.9)	47 (54.7)	0.262	18 (100.0)	47 (100.0)	–
NSCLC	22 (50.0)	27 (31.4)		–	–	–
RCC	1 (2.3)	3 (3.5)		–	–	–
GI	2 (4.5)	1 (1.2)		–	–	–
Breast	0 (0.0)	2 (2.3)		–	–	–
Head and Neck	0 (0.0)	1 (1.2)		–	–	–
Other	1 (2.3)	5 (5.8)		–	–	–
Unknown	13 (22.8)	4 (4.4)		0 (0.0)	0 (0.0)	
ICI Type (%)						
Combination	17 (40.5)	26 (30.2)	0.341	12 (70.6)	15 (31.9)	0.013
Monotherapy	25 (59.5)	60 (69.8)		5 (29.4)	32 (68.1)	
Unknown	15 (26.3)	4 (4.4)		1 (5.6)	0 (0.0)	
ICI Duration, median [IQR]	4.50 [1.6, 11.0]	3.00 [1.5, 4.3]	0.047	2.62 [0.9, 5.1]	2.25 [1.3, 3.9]	0.802
ICI Dur Unknown (%)	18 (31.6)	57 (63.3)		4 (22.2)	31 (66.0)	
Additional Therapy (%)						
Chemo	2 (7.7)	9 (20.0)	0.453	0 (0.0)	0 (0.0)	NA
RT	3 (11.5)	4 (8.9)		0 (0.0)	0 (0.0)	
Other	0 (0.0)	1 (2.2)		0 (0.0)	0 (0.0)	
None	21 (80.8)	31 (68.9)		6 (100.0)	14 (100.0)	
Unknown	31 (54.4)	45 (50.0)		12 (66.7)	33 (70.2)	
irAE Details						
Time to irAE, median [IQR]	4.00 [1.0, 8.3]	2.00 [1.0, 4.8]	0.091	1.71 [0.6, 7.8]	2.00 [1.0, 3.0]	0.988
Time to irAE Unknown(%)	0 (0.0)	0 (0.0)	–	0 (0.0)	0 (0.0)	–
irAE Duration	4.00 [3.0, 10.4]	1.38 [0.8, 4.1]	0.06	3.00 [2.3, 3.3]	0.98 [0.7, 2.6]	0.666
irAE Duration Missing	43 (75.4)	80 (88.9)	0.055	14 (77.8)	41 (87.2)	0.575
Primary irAE (%)						
Arthritis	17 (29.8)	17 (18.9)	0.053	2 (11.1)	7 (14.9)	0.004
Colitis	11 (19.3)	40 (44.4)		3 (16.7)	27 (57.4)	
Dermatologic	0 (0.0)	0 (0.0)		0 (0.0)	0 (0.0)	
Encephalitis	4 (7.0)	5 (5.6)		4 (22.2)	5 (10.6)	
Gastritis	0 (0.0)	0 (0.0)		0 (0.0)	0 (0.0)	
Hematologic	0 (0.0)	1 (1.1)		0 (0.0)	0 (0.0)	
Hepatitis	3 (5.3)	4 (4.4)		2 (11.1)	1 (2.1)	
Myositis/Myocarditis	3 (5.3)	1 (1.1)		0 (0.0)	0 (0.0)	
Nephritis	5 (8.8)	6 (6.7)		2 (11.1)	2 (4.3)	
Ocular	3 (5.3)	0 (0.0)		3 (16.7)	0 (0.0)	
Other	3 (5.3)	3 (3.3)		0 (0.0)	1 (2.1)	
Peripheral neuropathy	0 (0.0)	4 (4.4)		0 (0.0)	3 (6.4)	
Pneumonitis	5 (8.8)	6 (6.7)		2 (11.1)	0 (0.0)	
Vasculitis	3 (5.3)	3 (3.3)		0 (0.0)	1 (2.1)	
irAE Treatment Details						
Steroid Dur, med [IQR]	3.50 [1.6, 6.9]	3.25 [1.7, 4.0]	0.539	3.00 [0.2, 3.0]	1.17 [1.2, 3.0]	0.916
Steroid Dur missing	35 (61.4)	59 (65.6)	0.738	13 (72.2)	42 (89.4)	0.184
SSIA Duration, med [IQR]	–	1.88 [0.6, 5.4]	–	–	5.00 [1.4, 6.0]	–
SSIA Duration missing	–	32 (35.6)	–	–	29 (61.7)	–
SSIA Type (%)						
TNFI	–	50 (55.6)		–	29 (61.7)	
Other SSIA	–	28 (31.1)		–	10 (21.3)	
Immunomodulating agent	–	12 (13.3)	–	–	8 (17.0)	–
SSIA Regimen (%)						
Biologic + Conventional	–	3 (3.3)	–	–	0 (0.0)	-
Biologic only	–	59 (65.6)		–	33 (70.2)	–
Conventional only	–	12 (13.3)		–	6 (12.8)	
IVIg	–	11 (12.2)		–	8 (17.0)	
Other	–	5 (5.6)		–	0 (0.0)	

CS, Corticosteroid monotherapy; CS-SSIA, Corticosteroids and SSIA therapy; IQR, Interquartile range; NSCLC, Nonsmall cell lung cancer; RCC, Renal cell cancer; GI, Gastrointestinal; ICI, Immune checkpoint inhibitor; NA, Not applicable; Dur, Duration; RT, Radiotherapy; irAE, Immune-related adverse event; SSIA, Steroid-sparing immunosuppressant agent; TNFi, Tumor-necrosis factor alpha inhibitor; IVIg, Intravenous immunoglobulin.

*Landmark analysis:*

Only a proportion of patients were analyzed in the landmark analysis. Patients without information regarding either time to irAE, or time from irAE to steroids, or time from irAE to SSIA were excluded from the landmark analysis. These patients’ baseline characteristics are detailed in **Table 2B**. For these patients, we report patient characteristics evaluated *at the landmark time point* defined as 3 months after irAE to account for immortal time bias; thus, even though some patients had received SSIA after that time, they are included in the CS group. A total of 147 patients with any tumor type and 65 patients with melanoma were analyzed via landmark analysis. For any tumor type, 57 patients were in the CS cohort and 90 in CS-SSIA. Within the melanoma subgroup, 18 patients received CS monotherapy and 47 received a CS-SSIA combination. Missingness is detailed for the different variables as delineated in **Table 2B**. There was a difference in ICI duration between CS and CS-SSIA cohorts for any tumor type (Median 4.50 IQR (1.6, 11.0) and Median 3.0 IQR (1.5, 4.3), p=0.05) but not for patients in the melanoma subgroup (median 2.62 versus 2.25, p=0.8). In both groups, the most frequent irAE for which immunosuppression was started was colitis or arthritis. There was not a significant difference in either irAE duration or steroid duration between CS and CS-SSIA cohorts for any tumor type or melanoma.

#### Group-level data

A total of 22 studies had enough information to assess on a group-level basis, but not enough information to abstract data on an individual, patient level basis. Summary data are reflected in **Supplementary Table 6**. There were a total of 2558 patients reported in these studies. From these studies, 892 patients had received CS monotherapy whereas 1156 patients had received >= 1 SSIA(s) and were categorized as SSIA. There were a variety of malignancy types represented. Melanoma was the most common, being the focus of 12 studies with 1169 patients included (17, 19, 42, 57, 58, 64–68, 70, 71). One manuscript reported only patients with lung cancer and included n=87 patients (69). The remainder of publications included a mixture of malignancies (8, 20, 55, 56, 59–63). In terms of types of irAEs, 12 studies exclusively reported subjects with colitis (n=1437), one exclusively reported subjects with arthritis (n=147) and one with hepatitis (n=19). The remainder of the papers included a mixture of irAEs. Several studies had cohorts which received several different SSIAs or a combination of SSIAs.

### Association between SSIA use and tumor outcomes

#### Any SSIA

##### Individual-level data analysis for CS-SSIA vs CS

###### All *tumor types*

**Figure 2:**
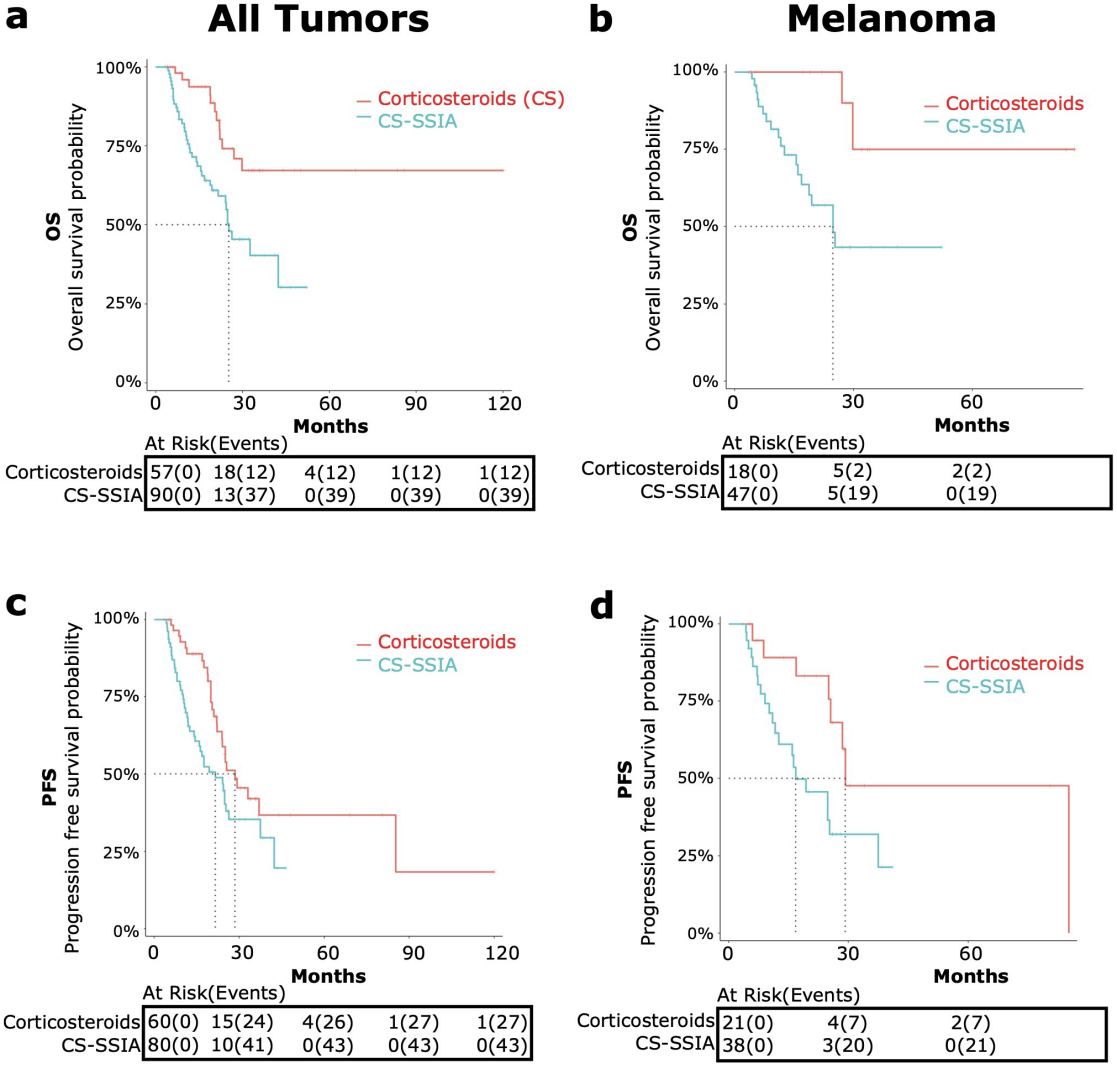
OS and PFS in patients treated with corticosteroids and steroid-sparing immunomodulating agents (CS-SSIA) versus corticosteroid monotherapy for immune-related adverse events treatment with adjustment for immortal time bias using 3 month landmark analysis. OS [top, **(A, B)**] and PFS [bottom, **(C, D)**] were compared between patients in the CS-SSIA (blue) vs corticosteroid alone (red) groups. Data was analyzed for all tumor types combined [left, **(A, C)**] and for melanoma alone [right, **(B, D)**].

When considering all tumor types, after landmark data processing was conducted to account for immortal time bias, OS data was available for n=90 CS-SSIA patients and n=57 CS patients (**Figure 2A**). PFS data was available for n=63 CS-SSIA patients and n=53 CS patients (**Figure 2C**). In the landmark analysis of patients with all tumor types, there was a significant higher risk of all-cause mortality for CS-SSIA when compared to CS (HR 2.75, 95% CI: 1.44-5.27, p<0.01) and a higher risk of progression for CS-SSIA vs CS (HR 1.75, 95% CI: 1.07-2.85, p=0.03).

The median OS for CS-SSIA was 25.3 months (95% CI 21.6-NA) and was not reached for CS (**Figure 2**). The median PFS for CS-SSIA was 21.6 months (95% CI: 15.9-37.5) and was 28.5 months for CS (95% CI: 23.9-NA) as displayed in **Figure 2**.

###### Melanoma

The subgroup of patients with melanoma was also analyzed separately, as melanoma was the most common tumor type. After landmark analysis, OS data was available for n=47 patients in the CS-SSIA cohort and for n=18 patients in the CS cohort (**Figure 2B**). For PFS, data was available for n=38 CS-SSIA patients and for n=21 CS patients (**Figure 2D**). There was a significant higher risk of all-cause mortality when comparing CS-SSIA vs CS group (HR 5.68, 95% CI: 1.31-24.67, p=0.02) and a higher risk of progression for CS-SSIA vs CS (HR 2.68, 95% CI: 1.12-6.40, p=0.03). The median OS for CS-SSIA was 24.8 months (95% CI: 16.8-NA) and was not reached for CS. The median PFS was 16.8 months for CS-SSIA (95% CI: 12.5-NA) and was 29.2 months for CS (95% CI: 25.5-NA).

OS and PFS for CS-SSIA versus CS for melanoma *without* adjusting for immortal time bias is provided in **Supplementary 5** in **Supplementary Data Sheet 1**.

##### Group-level data analysis for any SSIA

**Figure 3:**
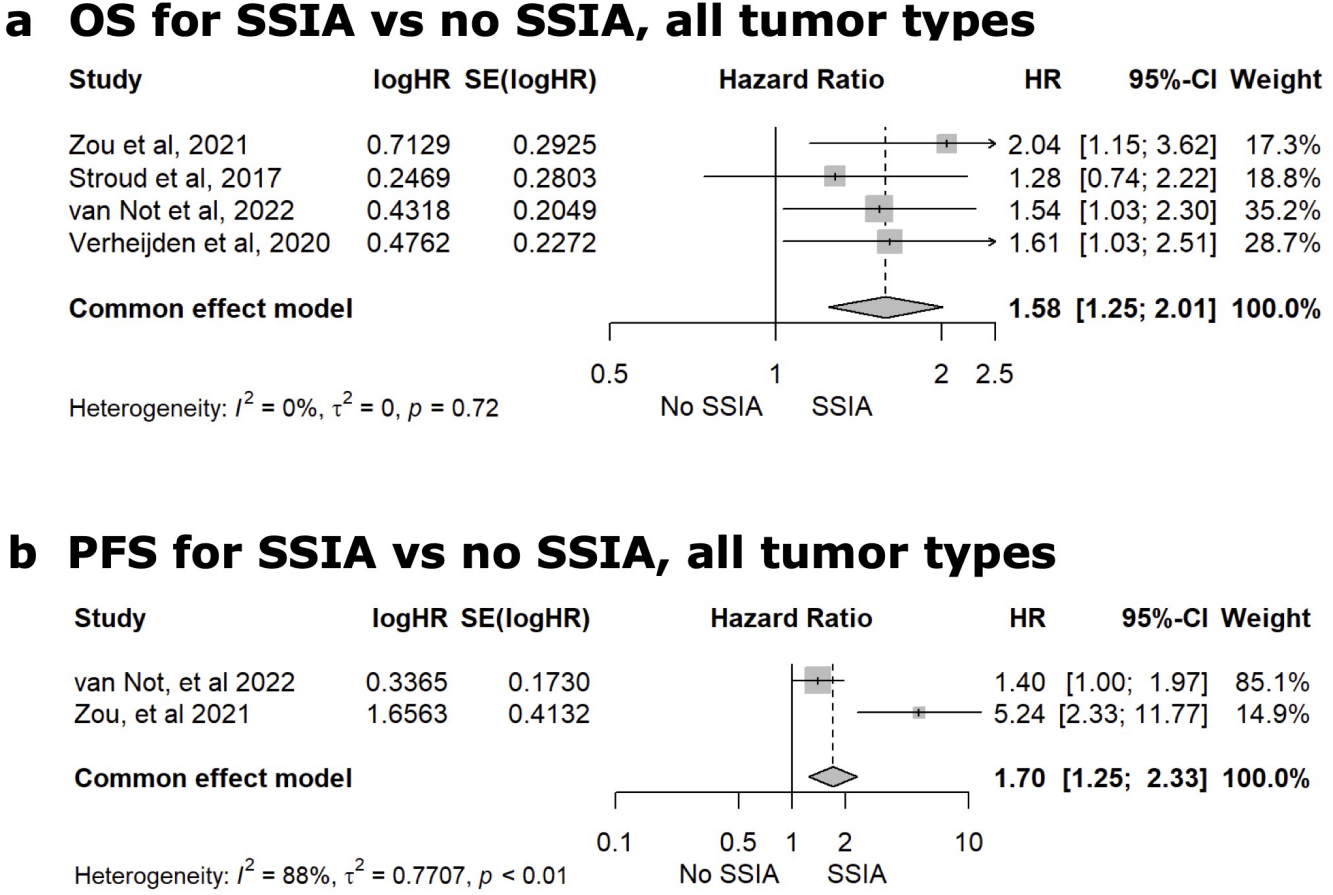
Meta-analysis comparing the adjusted hazard ratio (HR) for overall survival [OS, **(A)**] or progression-free survival [PFS **(B)**] after use of a steroid-sparing immunomodulating agent (SSIA) versus no SSIA for all tumor types.

Eighteen of the 22 studies reported tumor outcome results on OS and/or PFS. In most studies, the median OS or PFS and adjusted hazard ratio for OS or PFS needed to be estimated from the Kaplan Meier curve(s) available, and this data is reported in **Supplementary 6** in **Supplementary Data Sheet 1**. To be as accurate as possible in our meta-analysis and to optimize homogeneity, we only included the studies that specifically reported aHR with a 95% confidence interval on either OS or PFS in the meta-analysis and this ultimately resulted in 4 studies in this final meta-analysis for any SSIA versus no SSIA. Four studies reported on the adjusted hazard ratio for OS (19, 20, 69, 70). The meta-analysis for the pooled HR for all-cause mortality comparing SSIA versus no SSIA showed greater risk using SSIA (HR 1.58, 95%CI: 1.25-2.01, p<0.01), with a non-significant test of heterogeneity among the 4 studies (Q=1.35, p = 0.72) as reflected in **Figure 3A**. Only 2 studies reported the adjusted HR for risk of progression and meta-analysis showed a higher risk of progression for SSIA vs no SSIA (HR 1.70, 95%CI: 1.25-2.33, p<0.01), with a significant test of heterogeneity between the studies (Q=8.68, p < 0.01) as reflected in **Figure 3B**.

#### TNF-alpha inhibitors (TNFi)

##### Individual-level TNFi data analysis

**Figure 4:**
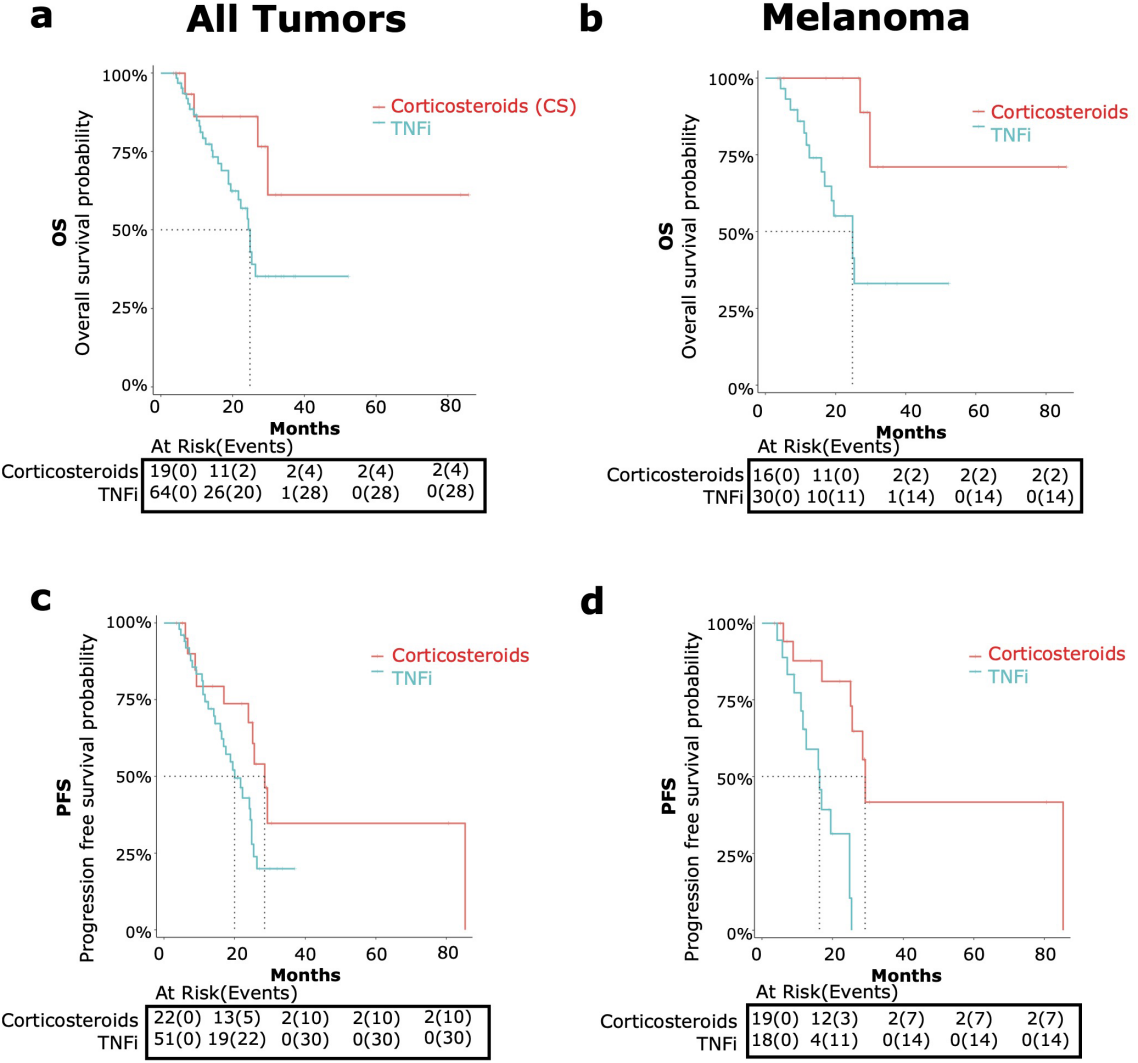
OS and PFS in patients receiving corticosteroids and tumor necrosis factor inhibitors (TNFi) versus corticosteroid (CS) monotherapy for immune-related adverse events (irAE) treatment with adjustment for immortal time bias using 3 month landmark analysis. [top, **(A, B)**] and PFS [bottom, **(C, D)**] were compared in patients receiving TNFi (blue) vs corticosteroid monotherapy (red). Data was analyzed for all tumor types combined [left graphs, **(A, C)**] and melanoma only [right graphs, **(B, D)**].

**Figure 5:**
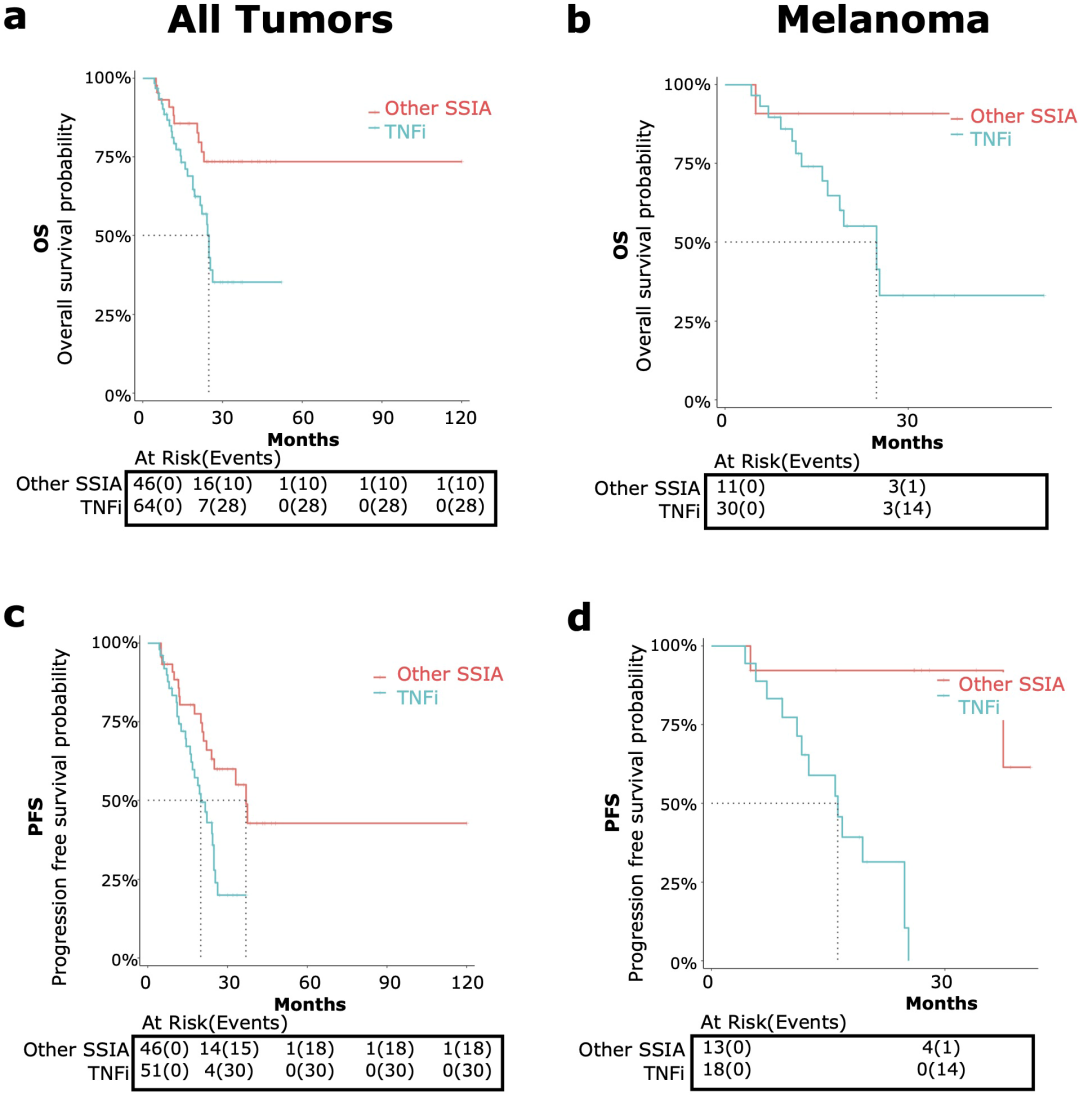
OS and PFS in patients receiving tumor necrosis factor inhibitors (TNFi) versus other steroid sparing immunomodulating agents (other SSIAs) for immune-related adverse events (irAE) treatment with adjustment for immortal time bias using 3 month landmark analysis. OS [top, **(A, B)**] and PFS {bottom, **(C, D)**] were compared in patients receiving TNFi (blue) vs other SSIAs (red). Data was analyzed for all tumor types combined [left graphs, **(A, C)**] and melanoma only [right graphs, **(B, D)**].

Landmark data processing was conducted to adjust for immortal time bias for patients with sufficient information for time to irAE, time from irAE to steroids, and time from irAE to TNFi. We conducted an individual-level data analysis comparing patients who received CS + TNF-alpha inhibitors (denoted as TNFi) versus CS monotherapy (CS) (**Figure 4**). The CS + TNFi group (TNFi) was also compared to CS + other SSIAs (Other SSIA) in **Figure 5**. OS and PFS without adjusting for immortal time bias for TNFi versus CS as well as TNFi versus other SSIAs in melanoma are provided in **Supplementary 7** in **Supplementary Data Sheet 1**.

###### TNFi versus CS

**All tumor types**

TNFis were the most common SSIA used (55.6%). When we compared the TNFi versus CS monotherapy groups among patients with any tumor type, we found a nearly significant higher risk of all-cause mortality (HR 2.88, 95% CI 1.00-8.35, p=0.05) (**Figure 4A**) and a trend toward a significant higher risk of progression (HR 1.96, 95% CI: 0.94-4.08, p=0.07) (**Figure 4C**).

The median OS for the TNFi group was 24.8 months (95% CI: 21.6-NA) and was not met for the CS group. The median TNFi PFS was 20 months (95% CI: 16.2-24.8) and 28.5 months (95% CI: 23.9-NA) for CS monotherapy.

**Melanoma**

TNFi were the most common SSIA for patients with melanoma also (44%). For these patients with melanoma, comparing TNFi versus CS monotherapy showed a significant higher risk of all-cause mortality (HR 6.46, 95% CI: 1.43-29.19, p=0.02) (**Figure 4B**) and a higher risk of progression (HR 7.49, 95% CI: 2.29-24.48, p<0.01) (**Figure 4D**).

The median OS in the TNFi group was 24.8 months (95% CI 16.8- NA) and was not reached for those receiving steroid monotherapy. The median PFS for the TNFi group was 16.2 months (95% CI: 11.6 – NA), which was notably shorter than the steroid monotherapy group PFS of 29.2 months (95% CI: 25.5 – NA).

###### TNFi versus Other SSIA

**All tumor types**

When comparing the use of TNFi versus other SSIAs, TNFi for irAE therapy showed a significant higher risk of all-cause mortality (HR 2.68, 95% CI: 1.30-5.56, p<0.01) (**Figure 5A**) and a higher risk of progression (HR 2.38, 95% CI: 1.29-4.39, p<0.01) for all tumor types (**Figure 5C**).

The median OS for patients receiving TNFi was 24.8 months (95% CI: 21.6-NA) and was not met for other SSIAs. The median PFS for patients receiving TNFi was 20 months (95% CI: 16.2-24.8) and for other SSIAs was 37 months (95% CI: 24.0-NA).

**Melanoma**

When compared to Other SSIAs, TNFi had a nearly significant higher risk of all-cause mortality (HR 6.96, 95% CI: 0.90-53.65, p=0.06) (**Figure 5B**) and a higher risk of progression (HR 21.5, 95% CI: 2.63-175.8, p<0.01) (**Figure 5D**).

The median OS in those receiving TNFi was 24.8 months (95% CI 16.8- NA) and was not reached for those in Other SSIA cohort. The median PFS for patients receiving TNFi was 16.2 months (95% CI: 11.6 – NA) and was not reached for Other SSIAs.

##### Group-level TNFi data analysis

**Figure 6:**
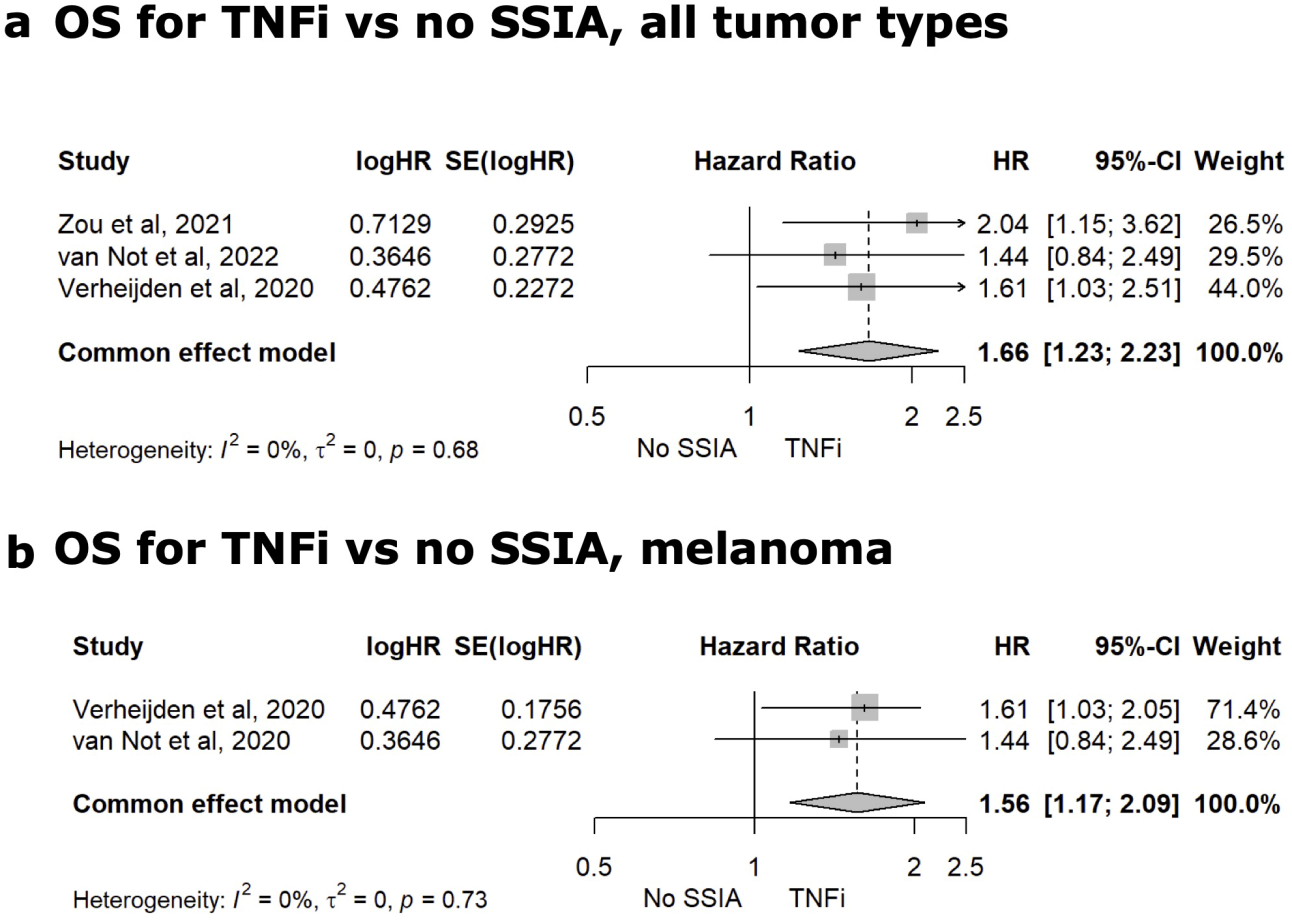
Meta-analysis for TNFi vs. No SSIA. Meta-analysis comparing the HR for OS for TNFi versus non SSIA in all tumor types **(A)** and for melanoma **(B)**.

Sixteen studies reported group-level data on patients receiving TNFi and **Supplementary 8** in **Supplementary Data Sheet 1** tabulates the median OS and PFS for TNFi vs no SSIAs as well as OS HR. Nine of the 16 studies were comparator studies and evaluated tumor outcomes for patients receiving TNFi for irAE therapy versus no SSIA (8, 19, 57, 61, 62, 66–68, 70). Only three studies had reported on HR for all-cause mortality and meta-analysis for this tumor outcome is reflected in **Figure 6A** with a statistically significant higher risk for TNFi versus no SSIA (HR 1.66, 95%CI: 1.23-2.23, p<0.01), with a non-significant test of heterogeneity between the studies (Q=0.78, p=0.68). For the cohort of patients with melanoma, meta-analysis showed a significantly higher risk of all-cause mortality for TNFi when compared to no SSIA (HR 1.56, 95% CI: 1.17-2.09, p<0.01), with a non-significant test of heterogeneity between the studies (Q=0.12, p = 0.73) as displayed in **Figure 6B**.

### Interleukin-6 receptor inhibitors (IL-6Ri)

Only 3 studies reported granular enough information for individual-level patient data regarding use of interleukin 6 receptor inhibitors (IL-6Ri), on a total of n=7 patients (**Supplementary 9** in **Supplementary Data Sheet 1**). The median duration of IL-6Ri therapy was 7.75 months. The most common indication for therapy was arthritis (n=4), followed by myocarditis (n=2). Progressive malignancy was reported in n=4 of the n=7 patients. From these studies with mixed tumor types, median OS was 21 months with a range of 6 to 48 months. Median PFS was 20.5 months with a range of 5 to 48 months. No robust studies provided group-level data via comparison of IL-6Ri versus no SSIAs or CS monotherapy and so no group-level data analyses could be conducted for IL-6Ri for irAE therapy.

## Discussion

This study is the first systematic literature review and meta-analysis evaluating the effect of SSIAs used for irAEs on OS and PFS on all tumor types, with a focus on melanoma. Our analyses raised concern for negative impact on tumor outcome after use of SSIAs and particularly TNFis for irAE treatment. After an individual-level landmark analysis accounting for immortal time bias, we found that OS and PFS were notably worse with CS-SSIA use versus CS monotherapy for all tumor types and for melanoma, and the group-level meta-analysis showed significantly higher risks of death and progression with SSIA use vs no SSIA. In our subanalysis of TNFi, there were significantly worse tumor outcomes when comparing CS with TNFi to CS monotherapy and TNFi to other SSIAs in both patients with melanoma and in all tumor types combined. These results should be interpreted with caution in light of inherent biases in conducting a literature review for retrospective, observational studies. However, given our findings, herewith we delve deeper into the role of TNFi within the ICI-treated tumor environment as well as the potential mechanistic advantage of looking into other SSIAs for irAE therapy such as IL-6 antagonists which have been suggested to enhance the anti-tumor efficacy of ICIs (23).

### TNF-alpha inhibitors

We report an increased risk of all-cause mortality and tumor progression for patients receiving TNFi for treatment of their irAEs, which may raise concern for their use. This was identified in both the aggregated analysis of individual patient level data and in the meta-analysis of group data. TNF-alpha blockade has been a ubiquitous strategy for management of irAEs, especially in colitis and arthritis. There have been mixed pre-clinical and retrospective data as to the potential impact of TNF blockade on tumor outcomes. Several groups have demonstrated that in murine models of melanoma and colon cancer, combining TNF blockade with ICI therapy abrogates the irAE and improves anti-cancer immune responses. One early clinical study of ICI associated colitis (ICI-Colitis) showed that adding one or two doses of infliximab to steroids was superior for control of colitis and did not compromise tumor response (66). A retrospective study of 93 patients with melanoma receiving infliximab for irAEs demonstrated no association with infliximab use and cancer progression; this study also assessed tumor outcomes for genitourinary cancers and saw longer PFS for patients using infliximab but results were not adjusted for immortal time bias (72). Another retrospective study looked at outcomes in patients with ICI-colitis requiring infliximab or vedolizumab who were subsequently rechallenged with ICIs (73). Survival outcomes were similar between patients with melanoma treated with ICIs who received concurrent infliximab or vedolizumab versus patients without selective immunosuppression (73). Of note, in this same study, ICI-colitis recurrence rates were lower in the group receiving maintenance infliximab and vedolizumab (73). A prospective Phase Ib trial that enrolled 14 patients with advanced and/or metastatic melanoma (stage IIIc/IV) combined ICI with TNF blockade (infliximab or certolizumab) and demonstrated favorable tumor outcomes: seven of seven evaluable patients achieved objective response with 4 CRs and 3 PRs (74). However, several retrospective studies have raised concern that TNFi agents may adversely affect tumor outcomes. A large study using data from the Dutch Melanoma Treatment registry showed that median OS was significantly longer in patients receiving steroids alone (n=235) compared to patients receiving either steroids + SSIAs (n=115) or steroids + TNFi (n=67) (19).

Bass et al. demonstrated that patients with ICI-arthritis treated with anti-TNF agents had a shorter time to cancer progression compared with those treated with methotrexate or tocilizumab (58). TNF blockade was also shown to be associated with worse overall survival compared to steroid monotherapy in a cohort of melanoma patients (70), and among patients with ICI-colitis compared to vedolizumab treatment (20). Our aggregated data from multiple studies comparing steroids alone to TNFi showed a correlation between worse OS and PFS with TNFi compared to steroids alone, especially among patients with melanoma. Among these 3 studies in the meta-analysis, there was relatively little heterogeneity in outcomes (I^2^ = 0%), also evidenced by a non-significant test of heterogeneity among the studies (Q=0.78, p = 0.68). Our analysis included a wide variety of treatment centers encompassing a broad time period and diverse practice patterns which strengthens the external validity of the analysis.

### IL-6 receptor antagonists

Our analysis of IL-6Ri was severely limited by a paucity of available reports, with only 3 studies reporting granular data on 7 total patients. Thus, a reliable comparison of the outcomes associated with IL-6Ri relative to other immunosuppressants could not be performed. Nonetheless, enthusiasm for use of these agents to mitigate irAEs is currently rising in the field, owing to encouraging pre-clinical data (23, 75). The pleiotropic cytokine IL-6 has a broad range of effects, including roles in inflammation and carcinogenesis (76, 77). Baseline elevated levels of IL-6 in serum and tissue, as well as a rise in serum IL-6 levels early in treatment have been correlated with poor tumor outcomes following ICI treatment (78–81). IL-6 underlies the pathogenesis of many autoimmune and inflammatory diseases by promoting the differentiation of naïve CD4+ T cells into Th17 cells, making IL-6 blockade an attractive treatment option for irAEs. In melanoma and colon cancer mouse models, Hailemichael et al. demonstrated that IL-6 blockade led to improved survival with an increased frequency of tumor-associated anti-CTLA4-induced CD8+ and CD4+ T effector cells and reduced Th17 CD4+ T cells, macrophages and myeloid cells (23). Several additional murine studies highlight the potential for improved anti-tumor response with combination therapy using anti-IL-6 with ICI, suggesting IL-6 blockade could decouple ICI anti-tumor immunity from toxicity (75). In this current systematic review, patient level data for IL-6 inhibition was limited such that the impact on tumor outcome could not be rigorously assessed. Limited clinical reports suggest that use of IL-6 inhibitors for irAEs may not attenuate ICI efficacy (34, 69, 82, 83). In the COLAR study (NCT03601611) evaluating the use of tocilizumab for ICI colitis or arthritis, 6 of 20 patients had progressive disease within the 24-week study period (84). Emerging results from prospective clinical trials suggest that combining tocilizumab, ipilimumab, and nivolumab may lead to a favorable cancer response with less toxicity (85–87). Results from a phase II trial with 70 patients showed that the upfront addition of tocilizumab to flipped-dose ipilimumab 1 mg/kg and nivolumab 3 mg/kg led to a 57% best ORR compared to the expected 47% in the historical control CheckMate-511 (86). This trial had a lower rate of grade 3-4 irAEs (22% with tocilizumab/ipilimumab/nivolumab compared to 34% in CheckMate-511). Another phase II trial combined ipilimumab 3 mg/kg and nivolumab 1 mg/kg with upfront tocilizumab. Tocilizumab was given either under a regular dosing regimen (162 mg subcutaneous bi-weekly for 6 doses; n=25) or a dose-dense regimen (162 mg subcutaneously weekly for 6 weeks, then biweekly for 6 weeks; n=10) (87). ORR was 56% at 12 weeks in the regular dose group and 70% in the dose dense group. The rate of grade 3-4 irAEs at 12 weeks was 44% in the regular dose group and 40% in the dose dense group. Whether IL-6 inhibition at initiation of ICI vs. at the onset of irAE is a better treatment strategy remains an open area of investigation. In addition, whether IL-6 inhibition may have differential tumor impacts depending on the timing of initiation, frequency of administration, or tumor type also remains an unanswered question.

### Prospective research into SSIA for irAE

Prospective clinical trials remain critically important to clarify if SSIA use is associated with worse tumor outcomes and if specific SSIAs have differing effects on tumor outcomes. Multiple prospective clinical trials are underway to assess the effectiveness and safety of SSIA(s) in treating various irAEs (NCT05335928, NCT04407247, NCT04375228, NCT04438382, NCT06037811, NCT04375228, NCT04810156). There are also multiple clinical trials to assess the benefit of IL6 antagonism in addition to ICI for cancer therapy with the aim of preventing or mitigating ICI toxicities (NCT04258150, NCT04940299, NCT03999749). A slightly different question being investigated is whether SSIAs used for pre-existing autoimmune disease affect tumor outcome after ICI therapy. A phase 1b study (AIM-NIVO) (NCT03816345) is currently enrolling patients with various autoimmune diseases and cancer to study overall safety and toxicities associated with use of nivolumab. Future research in this area will be aided by the development of specific ICD-10 codes for irAE diagnoses, an effort that is currently underway led by various irAE consortium groups (88). Systematic, prospective collection of data is critical to help mitigate some of the biases that are inherent in retrospective studies. However, until we have results and guidance from prospective studies, we recommend a greater emphasis on data sharing and uniform outcome reporting for future retrospective studies in this area.

### Missing data

Despite the depth of information that was available on the individual level, there was a considerable amount of missing data in regard to key variables needed for analysis in our study, summarized in **Supplementary 4** in **Supplementary Data Sheet 1**. Furthermore, there was a vast heterogeneity of data and outcome reporting between the different studies for tumor types, ICI regimen, irAE types, SSIA types and measurements for tumor outcomes. Most studies did not detail the relative start times of the irAE, systemic corticosteroids or SSIA(s) (including dose and duration) in relation to ICI initiation making it difficult to account for immortal time bias using time-varying covariates for the individual-level data. This level of missing details prevented us from being able to include more patients in the in-depth individual-level data analysis. For the group-level data, many studies and their respective Kaplan-Meier curves often had the following information missing: event-free at the start of each time interval, patients censored during the interval, patients at risk during the interval and number of events during each interval. Of note, we did not combine all the studies to evaluate as a whole within group-level data given the missingness and potential impact on outcome and results.

We realized the degree of data missingness through our review, highlighting the need for more granular data in individual papers and the need for enhanced data sharing efforts to enable meta-analyses. Foresight and standardization in data collection efforts will facilitate data aggregation efforts, so more granular research questions may be addressed. When asked, patients are largely in favor of data sharing efforts that have the potential to strengthen our knowledge base (89). Finally, we recommend that future retrospective studies reporting OS and PFS outcomes following irAEs present subanalyses that stratify by tumor type (even in the case of low numbers for each tumor type) as the expected OS and PFS differ by tumor type. Moreover, reporting HR and other tumor outcomes along with measures of uncertainty (SE or CI) would be highly beneficial even in the case when they are not significant. This will allow future meta-analysis efforts to identify important trends and distinctions.

### Limitations

It is important to acknowledge several limitations of our study. Most of the studies included in our analysis were retrospective and observational in nature, and therefore patients were not randomly distributed between the CS-SSIA and CS groups. The current clinical treatment guidelines for irAEs only recommend adding SSIA in patients with severe or refractory irAEs (3–6). As a result, patients in the CS only group may have had lower grade irAEs, lower CS doses, and a shorter overall CS treatment duration. One of the greatest limitations in this area of research is that it is difficult to untangle the impact on tumor outcome due to prolonged systemic CS exposure from the added impact of the SSIA(s) in many studies. We were also unable to distinguish between death due to tumor progression and death from irAEs, so the lower OS in the SSIA group could potentially also reflect increased mortality from higher grade irAEs. This underscores the need for prospective randomized controlled trials, to ensure uniformity of treatment exposures (i.e. CS only at a specified intensity and duration, vs. SSIA only at a specified intensity and duration). Randomized prospective trials will be needed to determine if worse outcomes are due to the biology of irAEs themselves or if it is truly the effect of the SSIA on the anti-tumor immune response. TNFi appear to perform worse than other SSIA; however, it is also important to note that TNFi is the most commonly used SSIA and the choice of SSIA may vary by toxicity which may confound these results. Subgroup analysis of the other SSIA groups was limited by the low numbers of studies and patients included.

Another limitation is the small sample size and heterogeneity across tumor types in our analysis. Although many other tumor types are represented in the data of these reports, they largely could not be disaggregated from the mixed populations for the purposes of our analysis. Because different tumor types have very distinctive and strong impacts on the expected OS and PFS, we performed pre-planned subgroup analyses of various tumor types but assessment of tumors other than melanoma were inadequately powered (e.g. renal cell carcinoma, NSCLC). Another limitation is publication bias, as the published reports likely represent a biased subset of the full population experience. In an attempt to mitigate some of this bias, we excluded case reports. We also excluded case series that “cherry-picked” cases or did not systematically identify all consecutive cases from the condition of interest. Because the patients in the SSIA cohorts had to have survived long enough to receive the SSIA treatment while those in the control groups did not have this requirement, our analysis was vulnerable to immortal time bias. To address this, we required at least a 3 month follow up after irAE and performed a landmark analysis using a landmark time of 3 months from irAE onset. Despite limitations, we were able to analyze considerable data representing patients across multiple institutions that reflect various SSIA practice patterns across the world.

## Conclusion

In our review, SSIAs showed worse outcomes compared to CS for irAE treatment on individual level and SSIAs fared worse compared to no SSIAs for irAEs on a group level for all tumor types and melanoma. Focusing on TNFi, patients with irAEs who received TNFi with CS consistently saw worse tumor outcomes compared to CS alone as well as compared to Other SSIAs with CS. Given multiple potential confounding factors inherent in retrospective research, these results should be interpreted with caution. Future studies should be developed with careful study design that minimizes heterogeneity in type and stage of tumor, as well as in CS and SSIA use (with respect to dose, duration, and timing). Until prospective clinical trials are published, robustly-conducted observational studies will have to inform expert opinion and guide clinical providers regarding which SSIAs are most safe and effective to use for second-line treatment for a particular irAEs and in the context of patient’s specific tumor type.

## Data Availability

The original contributions presented in the study are included in the article/**Supplementary Material**. Further inquiries can be directed to the corresponding author.
