# Supplementary material for: Impact of steroid-sparing immunosuppressive agents on tumor outcome in the context of cancer immunotherapy with highlight on melanoma: a systematic literature review and meta-analysis
Source: Front Immunol. 2024 Dec 16;15:1499478. doi: 10.3389/fimmu.2024.1499478 (PMC11682972; doi:10.3389/fimmu.2024.1499478)
Supplement: Supplementary file 1 [file DataSheet1.docx]

**SUPPLEMENTARY MATERIALS:**

[Supplement 1: Search Strategies](#supp1)

[Supplement 2a: Studies for Individual-level data extraction
Supplement 2b: Studies for Group-level data extraction](#supp2)

[Supplement 3: Quality assessment](#supp3)

[Supplement 4: Individual-level missing data, n(%)](#supp4)

[Supplement 5: OS and PFS after CS-SSIA vs. CS for melanoma *without adjusting for immortal time bias*](#supp5)

[Supplement 6: Group-level data for any SSIA](#supp6)

[Supplement 7a: OS and PFS for TNFi versus Steroids only *without adjusting for immortal time bias*Supplement 7b: TNFi versus Other SSIAs for melanoma *without adjusting for immortal time bias*](#supp7)

[Supplement 8: Group-level data for TNFi](#supp8)

[Supplement 9: Individual-level Data of Patients treated with IL6Ri](#supp9)

Supplement 1:

**Search Strategies**

| **PubMed** (NCBI) 1966-2023  848 references retrieved as of January 5, 2023. | (("Ipilimumab"[Mesh] OR Yervoy OR ipilimumab OR "MDX-010" OR "MDX010" OR "MDX-CTLA-4" OR "MDX-CTLA4" OR "pembrolizumab"[Supplementary Concept] OR Keytruda OR lambrolizumab OR pembrolizumab OR "SCH-900475" OR "SCH900475" OR "MK-3475" OR "MK3475" OR "Nivolumab"[Mesh] OR Opdivo OR nivolumab OR "BMS-936558" OR "BMS936558" OR "MDX-1106" OR "MDX1106" OR "ONO-4538" OR "ONO4538" OR "atezolizumab"[Supplementary Concept] OR "MDPL-3280A" OR "MDPL3280A" OR "RG-7446" OR "RG7446" OR Tecentriq OR atezolizumab OR "avelumab"[Supplementary Concept] OR Bavencio OR avelumab OR "msb-0010718c" OR "msb0010718c" OR "cemiplimab"[Supplementary Concept] OR Libtayo OR cemiplimab OR "REGN-2810" OR "REGN2810" OR "durvalumab"[Supplementary Concept] OR Imfinzi OR durvalumab OR "MEDI-4736" OR "MEDI4736" OR "dostarlimab-gxly"[Supplementary Concept] OR Jemperli OR dostarlimab OR "GSK-4057190" OR "GSK4057190" OR "TSR-042" OR "TSR042" OR "camrelizumab"[Supplementary Concept] OR AiRuiKa OR camrelizumab OR "SHR-1210" OR "SHR1210" OR "tremelimumab"[Supplementary Concept] OR ticilimumab OR tremelimumab OR "CP-675*" OR "CP675*" OR "Immune Checkpoint Inhibitors"[Mesh] OR "Programmed Cell Death 1 Receptor/antagonists and inhibitors"[Mesh] OR "CTLA-4 Antigen/antagonists and inhibitors"[Mesh] OR "B7-H1 Antigen/antagonists and inhibitors"[Mesh] OR "checkpoint inhibit*" OR "checkpoint block*" OR "checkpoint antagonist*" OR "PD-L1 inhibitor*" OR "PDL1 inhibitor*" OR "Programmed Death Ligand 1 Inhibitor*" OR "Programmed Cell Death Ligand 1 Inhibitor*" OR "PD-L1 antibod*" OR "PDL1 antibod*" OR "Programmed Death Ligand 1 antibod*" OR "Programmed Cell Death Ligand 1 antibod*" OR "anti-PD-L1" OR "anti-PDL1" OR "Programmed Death Ligand 1 antagonist*" OR "Programmed Cell Death Ligand 1 antagonist*" OR "PD-L1 antagonist*" OR "PDL1 antagonist*" OR "Programmed Death Ligand 1 block*" OR "Programmed Cell Death Ligand 1 block*" OR "PD-L1 block*" OR "PDL1 block*" OR "PD-1 Inhibitor*" OR "PD1 inhibitor*" OR "Programmed Cell Death Protein 1 Inhibitor*" OR "PD-1 antibod*" OR "PD1 antibod*" OR "Programmed Cell Death Protein 1 Antibod*" OR "PD-1-PD-L1 Block*" OR "anti-PD-1" OR "anti-PD1" OR "anti-programmed death" OR "antiprogrammed death" OR "PD-1 antagonist*" OR "PD1 antagonist*" OR "Programmed Cell Death Protein 1 antagonist*" OR "PD-1 receptor block*" OR "PD1 receptor block*" OR "PD-1 block*" OR "PD1 block*" OR "programmed cell death 1 receptor block*" OR "PD-1 receptor antagonist*" OR "PD1 receptor antagonist*" OR "programmed cell death 1 receptor antagonist*" OR "PD-1 receptor antibod*" OR "PD1 receptor antibod*" OR "programmed cell death 1 receptor antibod*" OR "CTLA-4 Inhibitor*" OR "CTLA4 inhibitor*" OR "Cytotoxic T-Lymphocyte-Associated Protein 4 Inhibitor*" OR "cytotoxic T lymphocyte associated antigen 4 inhibitor*" OR "cytotoxic T-lymphocyte antigen 4 inhibitor*" OR "CTLA-4 antibod*" OR "CTLA4 antibod*" OR "Cytotoxic T-Lymphocyte-Associated Protein 4 Antibod*" OR "cytotoxic T lymphocyte-associated antigen 4 antibod*" OR "cytotoxic T lymphocyte antigen 4 antibod*" OR "anti-CTLA-4" OR "anti-CTLA4" OR "anti-Cytotoxic T-Lymphocyte-Associated Protein 4" OR "anti-cytotoxic T lymphocyte associated antigen*" OR "anti-cytotoxic T-lymphocyte antigen 4" OR "CTLA-4 antagonist*" OR "CTLA4 antagonist*" OR "CTLA-4 antigen antagonist*" OR "Cytotoxic T-Lymphocyte-Associated Protein 4 antagonist*" OR "cytotoxic T lymphocyte–associated antigen 4 antagonist*" OR "cytotoxic T lymphocyte antigen 4 antagonist*" OR "CTLA-4 antigen block*" OR "Cytotoxic T-Lymphocyte-Associated Protein 4 block*" OR **"**cytotoxic T lymphocyte–associated antigen 4 block*" OR "cytotoxic T lymphocyte antigen 4 block*" OR "CTLA-4 block*" OR "CTLA4 block*")  AND  ("tocilizumab"[Supplementary Concept] OR Actemra OR Roactemra OR Atlizumab OR tocilizumab OR "sarilumab"[Supplementary Concept] OR Kevzara OR sarilumab OR "siltuximab"[Supplementary Concept] OR Sylvant OR siltuximab OR "Infliximab"[Mesh] OR Remicade OR Renflexis OR Inflectra OR Avakine OR Remsima OR Avsola OR Flixabi OR Renflexis OR infliximab OR "infliximab-dyyb" OR "Adalimumab"[Mesh] OR Amjevita OR Cyltezo OR Humira OR Trudexa OR adalimumab OR "Etanercept"[Mesh] OR Enbrel OR Elrezi OR etanercept OR "golimumab"[Supplementary Concept] OR Simponi OR golimumab OR "Certolizumab Pegol"[Mesh] OR Cimzia OR certolizumab OR "Rituximab"[Mesh] OR Mabthera OR Rituxan OR rituximab OR "belimumab" [Supplementary Concept] OR LymphoStat OR Benlysta OR belimumab OR "Interleukin 1 Receptor Antagonist Protein"[Mesh] OR Antril OR Kineret OR Anakinra OR "canakinumab"[Supplementary Concept] OR Ilaris OR canakinumab OR "Abatacept"[Mesh] OR Belatacept OR Orencia OR Nulojix OR abatacept OR "Ustekinumab"[Mesh] OR Stelara OR ustekinumab OR "secukinumab"[Supplementary Concept] OR Cosentyx OR secukinumab OR "ixekizumab"[Supplementary Concept] OR Taltz OR ixekizumab OR "guselkumab" [Supplementary Concept] OR Tremfya OR guselkumab OR "tildrakizumab"[Supplementary Concept] OR Ilumya OR tildrakizumab OR "risankizumab"[Supplementary Concept] OR skyrizi OR risankizumab OR "vedolizumab"[Supplementary Concept] OR Entyvio OR vedolizumab OR "Omalizumab"[Mesh] OR Xolair OR omalizumab OR "mepolizumab"[Supplementary Concept] OR Bosatria OR Nucala OR mepolizumab OR "benralizumab" [Supplementary Concept] OR Fasenra OR benralizumab OR "Sirolimus"[Mesh] OR rapamycin OR Rapamune OR sirolimus OR "Everolimus"[Mesh] OR Afinitor OR Zortress OR Certican OR everolimus OR "temsirolimus" [Supplementary Concept] OR Torisel OR temsirolimus OR "Alemtuzumab"[Mesh] OR Campath OR Lemtrada OR alemtuzumab OR "Mitoxantrone"[Mesh] OR Novantrone OR mitoxantrone OR "ocrelizumab" [Supplementary Concept] OR Ocrevus OR ocrelizumab OR "Natalizumab"[Mesh] OR Tysabri OR natalizumab OR "Mycophenolic Acid"[Mesh] OR mycophenolate OR Cellcept OR Myfortic OR "mycophenolic acid" OR "Azathioprine"[Mesh] OR Imurel OR Imuran OR Immuran OR azathioprine OR "Methotrexate"[Mesh] OR Amethopterin OR Mexate OR methotrexate OR "Hydroxychloroquine"[Mesh] OR Oxychlorochin OR Oxychloroquine OR Hydroxychlorochin OR Plaquenil OR hydroxychloroquine OR "Tacrolimus"[Mesh] OR Prograf OR Prograft OR tacrolimus OR "Cyclosporine"[Mesh] OR Neoral OR Sandimmun OR Sandimmune OR cyclosporin OR cyclosporine OR "Leflunomide"[Mesh] OR Arava OR leflunomide OR "Sulfasalazine"[Mesh] OR Sulfazine OR Azulfidine OR Salicylazosulfapyridine OR Sulphasalazine OR Salazosulfapyridine OR Colo-Pleon OR Pleon OR sulfasalazine OR "tofacitinib" [Supplementary Concept] OR Xeljanz OR tofacitinib OR "baricitinib" [Supplementary Concept] OR Olumiant OR baricitinib OR "upadacitinib"[Supplementary Concept] OR Rinvoq OR upadacitinib OR "ruxolitinib" [Supplementary Concept] OR Jakafi OR ruxolitinib OR "Immunoglobulins, Intravenous"[Mesh] OR "intravenous immunoglobulin*" OR "IV immunoglobulin*" OR "IV gammaglobulin" OR "IVIG" OR Flebogamma OR Gamunex OR "Globulin-N" OR Intraglobin OR Gammagard OR Gamimune OR Gamimmune OR Privigen OR Sandoglobulin OR Venoglobulin OR Venimmune OR Iveegam OR Alphaglobin OR Endobulin OR Gammonativ OR "Plasmapheresis"[Mesh] OR "therapeutic Immunoadsorption" OR "therapeutic plasma adsorption" OR plasmapheresis OR "apremilast" [Supplementary Concept] OR Otezla OR apremilast OR "Cyclophosphamide"[Mesh] OR Sendoxan OR Cytoxan OR Endoxan OR Neosar OR Procytox OR cyclophosphamide OR "Antilymphocyte Serum"[Mesh] OR "anti-thymocyte globulin*" OR "antithymocyte globulin*" OR "anti-lymphocyte serum" OR "antilymphocyte serum" OR "antilymphocyte globulin*" OR "anti-lymphocyte globulin*" OR "anti-lymphocyte immunoglobulin*" OR "antilymphocyte immunoglobulin*" OR Pressimmune OR ATGAM)  AND  ("Clinical Study" [Publication Type] OR "Clinical Trial" [Publication Type] OR "Observational Study" [Publication Type] OR "Comparative Study" [Publication Type] OR "Multicenter Study" [Publication Type] OR "Cohort Studies"[Mesh] OR "Retrospective Studies"[Mesh] OR "Case-Control Studies"[Mesh] OR "Follow-Up Studies"[Mesh] OR "Clinical Studies as Topic"[Mesh] OR "Controlled Clinical Trials as Topic"[Mesh] OR "Multicenter Studies as Topic"[Mesh] OR "Control Groups"[Mesh:noexp] OR "control group*"[tw] OR "clinical stud*"[tw] OR "clinical trial*"[tw] OR retrospective*[tw] OR prospective*[tw] OR observational[tw] OR "comparative stud*"[tw] OR "follow up"[tw] OR longitudinal*[tw] OR multicenter[tw] OR cohort[tw] OR cohorts[tw] OR randomised[tw] OR randomized[tw] OR "case series"[tw] OR "record review*"[tw])  AND  "english"[Language])  NOT ("animals"[mesh] NOT "humans"[mesh])  NOT (("Review"[pt] NOT ("record review*"[tw] OR “records review*”[tw] OR “case series”[tw])) OR "Letter" [Publication Type] OR "editorial"[Publication Type] OR “case report*”[ti] OR “reported case*”[ti]) |
| --- | --- |
| **Embase** (Elsevier) 1947-2023  3,860 references retrieved as of January 5, 2023. | (("ipilimumab"/exp OR yervoy OR ipilimumab OR "mdx-010" OR "mdx010" OR "mdx-ctla-4" OR "mdx-ctla4" OR "pembrolizumab"/exp OR keytruda OR lambrolizumab OR pembrolizumab OR "sch-900475" OR "sch900475" OR "mk-3475" OR "mk3475" OR 'nivolumab"/exp OR opdivo OR nivolumab OR "bms-936558" OR "bms936558" OR "mdx-1106" OR "mdx1106" OR "ono-4538" OR "ono4538" OR "atezolizumab"/exp OR "mdpl-3280a" OR "mdpl3280a" OR "rg-7446" OR "rg7446" OR tecentriq OR atezolizumab OR "avelumab"/exp OR bavencio OR avelumab OR "msb-0010718c" OR "msb0010718c" OR "cemiplimab"/exp OR libtayo OR cemiplimab OR "regn-2810" OR "regn2810" OR "durvalumab"/exp OR imfinzi OR durvalumab OR "medi-4736" OR "medi4736" OR "dostarlimab"/exp OR jemperli OR dostarlimab OR "gsk-4057190" OR "gsk4057190" OR "tsr-042" OR "tsr042" OR "camrelizumab"/exp OR airuika OR camrelizumab OR "shr-1210" OR "shr1210" OR "ticilimumab"/exp OR ticilimumab OR tremelimumab OR "cp-675*" OR "cp675*" OR "immune checkpoint inhibitor"/exp OR "cytotoxic t lymphocyte antigen 4 antibody"/exp OR "checkpoint inhibit*" OR "checkpoint block*" OR "checkpoint antagonist*" OR "pd-l1 inhibitor*" OR "pdl1 inhibitor*" OR "programmed death ligand 1 inhibitor*" OR "programmed cell death ligand 1 inhibitor*" OR "pd-l1 antibod*" OR "pdl1 antibod*" OR "programmed death ligand 1 antibod*" OR "programmed cell death ligand 1 antibod*" OR "anti-pd-l1" OR "anti-pdl1" OR "programmed death ligand 1 antagonist*" OR "programmed cell death ligand 1 antagonist*" OR "pd-l1 antagonist*" OR "pdl1 antagonist*" OR "programmed death ligand 1 block*" OR "programmed cell death ligand 1 block*" OR "pd-l1 block*" OR "pdl1 block*" OR "pd-1 inhibitor*" OR "pd1 inhibitor*" OR "programmed cell death protein 1 inhibitor*" OR "pd-1 antibod*" OR "pd1 antibod*" OR "programmed cell death protein 1 antibod*" OR "pd-1-pd-l1 block*" OR "anti-pd-1" OR "anti-pd1" OR "anti-programmed death" OR "antiprogrammed death" OR "pd-1 antagonist*" OR "pd1 antagonist*" OR "programmed cell death protein 1 antagonist*" OR "pd-1 receptor block*" OR "pd1 receptor block*" OR "pd-1 block*" OR "pd1 block*" OR "programmed cell death 1 receptor block*" OR "pd-1 receptor antagonist*" OR "pd1 receptor antagonist*" OR "programmed cell death 1 receptor antagonist*" OR "pd-1 receptor antibod*" OR "pd1 receptor antibod*" OR "programmed cell death 1 receptor antibod*" OR "ctla-4 inhibitor*" OR "ctla4 inhibitor*" OR "cytotoxic t-lymphocyte-associated protein 4 inhibitor*" OR "cytotoxic t lymphocyte associated antigen 4 inhibitor*" OR "cytotoxic t-lymphocyte antigen 4 inhibitor*" OR "ctla-4 antibod*" OR "ctla4 antibod*" OR "cytotoxic t-lymphocyte-associated protein 4 antibod*" OR "cytotoxic t lymphocyte-associated antigen 4 antibod*" OR "cytotoxic t lymphocyte antigen 4 antibod*" OR "anti-ctla-4" OR "anti-ctla4" OR "anti-cytotoxic t-lymphocyte-associated protein 4" OR "anti-cytotoxic t lymphocyte associated antigen*" OR "anti-cytotoxic t-lymphocyte antigen 4" OR "ctla-4 antagonist*" OR "ctla4 antagonist*" OR "ctla-4 antigen antagonist*" OR "cytotoxic t-lymphocyte-associated protein 4 antagonist*" OR "cytotoxic t lymphocyte–associated antigen 4 antagonist*" OR "cytotoxic t lymphocyte antigen 4 antagonist*" OR "ctla-4 antigen block*" OR "cytotoxic t-lymphocyte-associated protein 4 block*" OR "cytotoxic t lymphocyte–associated antigen 4 block*" OR "cytotoxic t lymphocyte antigen 4 block*" OR "ctla-4 block*" OR "ctla4 block*")  AND  ("tocilizumab"/exp OR Actemra OR Roactemra OR Atlizumab OR tocilizumab OR "sarilumab"/exp OR Kevzara OR sarilumab OR "siltuximab"/exp OR Sylvant OR siltuximab OR "infliximab"/exp OR Remicade OR Renflexis OR Inflectra OR Avakine OR Remsima OR Avsola OR Flixabi OR Renflexis OR infliximab OR "infliximab-dyyb" OR "adalimumab"/exp OR Amjevita OR Cyltezo OR Humira OR Trudexa OR adalimumab OR "etanercept"/exp OR Enbrel OR Elrezi OR etanercept OR "golimumab"/exp OR Simponi OR golimumab OR "certolizumab pegol"/exp OR Cimzia OR certolizumab OR "rituximab"/exp OR Mabthera OR Rituxan OR rituximab OR "belimumab"/exp OR LymphoStat OR Benlysta OR belimumab OR "anakinra"/exp OR Antril OR Kineret OR anakinra OR "canakinumab"/exp OR Ilaris OR canakinumab OR "abatacept"/exp OR Belatacept OR Orencia OR Nulojix OR abatacept OR "ustekinumab"/exp OR Stelara OR ustekinumab OR "secukinumab"/exp OR Cosentyx OR secukinumab OR "ixekizumab"/exp OR Taltz OR ixekizumab OR "guselkumab"/exp OR Tremfya OR guselkumab OR "tildrakizumab"/exp OR Ilumya OR tildrakizumab OR "Risankizumab"/exp OR skyrizi OR Risankizumab OR "vedolizumab"/exp OR Entyvio OR vedolizumab OR "omalizumab"/exp OR Xolair OR omalizumab OR "mepolizumab"/exp OR Bosatria OR Nucala OR mepolizumab OR "benralizumab"/exp OR Fasenra OR benralizumab OR "rapamycin"/exp OR rapamycin:ti,ab OR Rapamune OR sirolimus OR "everolimus"/exp OR Afinitor OR Zortress OR Certican OR everolimus OR "temsirolimus"/exp OR Torisel OR temsirolimus OR "alemtuzumab"/exp OR Campath OR Lemtrada OR alemtuzumab OR "mitoxantrone"/exp OR Novantrone OR mitoxantrone OR "ocrelizumab"/exp OR Ocrevus OR ocrelizumab OR "natalizumab"/exp OR Tysabri OR natalizumab OR "mycophenolic acid"/exp OR mycophenolate OR Cellcept OR Myfortic OR "mycophenolic acid" OR "azathioprine"/exp OR Imurel OR Imuran OR Immuran OR azathioprine OR "methotrexate"/exp OR Amethopterin OR Mexate OR methotrexate OR "hydroxychloroquine"/exp OR Oxychlorochin OR Oxychloroquine OR Hydroxychlorochin OR Plaquenil OR hydroxychloroquine OR "tacrolimus"/exp OR Prograf OR Prograft OR tacrolimus OR "cyclosporine"/exp OR Neoral OR Sandimmun OR Sandimmune OR cyclosporin OR cyclosporine OR "leflunomide"/exp OR Arava OR leflunomide OR "salazosulfapyridine"/exp OR Sulfazine OR Azulfidine OR Salicylazosulfapyridine OR Sulphasalazine OR Salazosulfapyridine OR Colo-Pleon OR Pleon OR sulfasalazine OR "tofacitinib"/exp OR Xeljanz OR tofacitinib OR "baricitinib"/exp OR Olumiant OR baricitinib OR "upadacitinib"/exp OR Rinvoq OR upadacitinib OR "ruxolitinib"/exp OR Jakafi OR ruxolitinib OR "immunoglobulin"/exp/dd_iv OR "intravenous immunoglobulin*" OR "IV immunoglobulin*" OR "IV gammaglobulin" OR "IVIG" OR Flebogamma OR Gamunex OR "Globulin-N" OR Intraglobin OR Gammagard OR Gamimune OR Gamimmune OR Privigen OR Sandoglobulin OR Venoglobulin OR Venimmune OR Iveegam OR Alphaglobin OR Endobulin OR Gammonativ OR "upadacitinib"/exp OR Rinvoq OR upadacitinib OR "ruxolitinib"/exp OR Jakafi OR ruxolitinib OR "plasmapheresis"/exp OR "therapeutic Immunoadsorption" OR "therapeutic plasma adsorption" OR plasmapheresis OR "apremilast"/exp OR Otezla OR apremilast OR "cyclophosphamide"/exp OR Sendoxan OR Cytoxan OR Endoxan OR Neosar OR Procytox OR cyclophosphamide OR "thymocyte antibody"/exp OR "anti-thymocyte globulin*" OR "antithymocyte globulin*" OR "anti-lymphocyte serum" OR "antilymphocyte serum" OR "antilymphocyte globulin*" OR "anti-lymphocyte globulin*" OR "anti-lymphocyte immunoglobulin*" OR "antilymphocyte immunoglobulin*" OR Pressimmune OR ATGAM)  AND  ("case control study"/exp OR "clinical article"/exp OR "clinical trial"/exp OR "family study"/exp OR "intervention study"/exp OR "longitudinal study"/exp OR "major clinical study"/exp OR "open study"/exp OR "postmarketing surveillance"/exp OR "prospective study"/exp OR "retrospective study"/exp OR "clinical study"/de OR "comparative study"/de OR "comparative effectiveness"/exp OR "dosage schedule comparison"/exp OR "drug comparison"/exp OR "drug dosage form comparison"/exp OR "drug dose comparison"/exp OR "cohort analysis"/exp OR "controlled study"/exp OR "clinical trial (topic)"/exp OR "medical record review"/exp OR "control group*":ti,ab,kw OR "case series":ti,ab,kw OR "clinical stud*":ti,ab,kw OR "clinical trial*":ti,ab,kw OR retrospective*:ti,ab,kw OR prospective*:ti,ab,kw OR observational:ti,ab,kw OR "comparative stud*":ti,ab,kw OR "follow up":ti,ab,kw OR longitudinal*:ti,ab,kw OR multicenter:ti,ab,kw OR cohort:ti,ab,kw OR cohorts:ti,ab,kw OR randomised:ti,ab,kw OR randomized:ti,ab,kw OR "record review*":ti,ab,kw)  AND  "English":la)  NOT  ([animals]/lim NOT [humans]/lim)  NOT  ('conference abstract':it OR (review:it NOT ('case series' OR 'record review' OR ‘records review’)) OR 'single-case study'/exp OR 'letter'/exp OR 'editorial'/exp OR ‘case report*’:ti OR ‘reported case*’:ti) |
| **Web of Science Core Collection** (Clarivate)  Science Citation Index Expanded 1900-2023;  Social Sciences Citation Index 1975-2023;  Arts & Humanities Citation Index 1975-2023;  Emerging Sources Citation Index 2018-2023.  793 references retrieved as of January 5, 2023. | TS=(ipilimumab OR yervoy OR "mdx-010" OR "mdx010" OR "mdx-ctla-4" OR "mdx-ctla4" OR pembrolizumab OR keytruda OR lambrolizumab OR "sch-900475" OR "sch900475" OR "mk-3475" OR "mk3475" OR nivolumab OR opdivo OR "bms-936558" OR "bms936558" OR "mdx-1106" OR "mdx1106" OR "ono-4538" OR "ono4538" OR atezolizumab OR "mdpl-3280a" OR "mdpl3280a" OR "rg-7446" OR "rg7446" OR tecentriq OR avelumab OR bavencio OR "msb-0010718c" OR "msb0010718c" OR cemiplimab OR libtayo OR "regn-2810" OR "regn2810" OR durvalumab OR imfinzi OR "medi-4736" OR "medi4736" OR dostarlimab OR jemperli OR "gsk-4057190" OR "gsk4057190" OR "tsr-042" OR "tsr042" OR camrelizumab OR airuika OR "shr-1210" OR "shr1210" OR ticilimumab OR tremelimumab OR "cp-675*" OR "cp675*" OR "checkpoint inhibit*" OR "checkpoint block*" OR "checkpoint antagonist*" OR "pd-l1 inhibitor*" OR "pdl1 inhibitor*" OR "programmed death ligand 1 inhibitor*" OR "programmed cell death ligand 1 inhibitor*" OR "pd-l1 antibod*" OR "pdl1 antibod*" OR "programmed death ligand 1 antibod*" OR "programmed cell death ligand 1 antibod*" OR "anti-pd-l1" OR "anti-pdl1" OR "programmed death ligand 1 antagonist*" OR "programmed cell death ligand 1 antagonist*" OR "pd-l1 antagonist*" OR "pdl1 antagonist*" OR "programmed death ligand 1 block*" OR "programmed cell death ligand 1 block*" OR "pd-l1 block*" OR "pdl1 block*" OR "pd-1 inhibitor*" OR "pd1 inhibitor*" OR "programmed cell death protein 1 inhibitor*" OR "pd-1 antibod*" OR "pd1 antibod*" OR "programmed cell death protein 1 antibod*" OR "pd-1-pd-l1 block*" OR "anti-pd-1" OR "anti-pd1" OR "anti-programmed death" OR "antiprogrammed death" OR "pd-1 antagonist*" OR "pd1 antagonist*" OR "programmed cell death protein 1 antagonist*" OR "pd-1 receptor block*" OR "pd1 receptor block*" OR "pd-1 block*" OR "pd1 block*" OR "programmed cell death 1 receptor block*" OR "pd-1 receptor antagonist*" OR "pd1 receptor antagonist*" OR "programmed cell death 1 receptor antagonist*" OR "pd-1 receptor antibod*" OR "pd1 receptor antibod*" OR "programmed cell death 1 receptor antibod*" OR "ctla-4 inhibitor*" OR "ctla4 inhibitor*" OR "cytotoxic t-lymphocyte-associated protein 4 inhibitor*" OR "cytotoxic t lymphocyte associated antigen 4 inhibitor*" OR "cytotoxic t-lymphocyte antigen 4 inhibitor*" OR "ctla-4 antibod*" OR "ctla4 antibod*" OR "cytotoxic t-lymphocyte-associated protein 4 antibod*" OR "cytotoxic t lymphocyte-associated antigen 4 antibod*" OR "cytotoxic t lymphocyte antigen 4 antibod*" OR "anti-ctla-4" OR "anti-ctla4" OR "anti-cytotoxic t-lymphocyte-associated protein 4" OR "anti-cytotoxic t lymphocyte associated antigen*" OR "anti-cytotoxic t-lymphocyte antigen 4" OR "ctla-4 antagonist*" OR "ctla4 antagonist*" OR "ctla-4 antigen antagonist*" OR "cytotoxic t-lymphocyte-associated protein 4 antagonist*" OR "cytotoxic t lymphocyte–associated antigen 4 antagonist*" OR "cytotoxic t lymphocyte antigen 4 antagonist*" OR "ctla-4 antigen block*" OR "cytotoxic t-lymphocyte-associated protein 4 block*" OR "cytotoxic t lymphocyte–associated antigen 4 block*" OR "cytotoxic t lymphocyte antigen 4 block*" OR "ctla-4 block*" OR "ctla4 block*")  AND  TS=(tocilizumab OR Actemra OR Roactemra OR Atlizumab OR sarilumab OR Kevzara OR siltuximab OR Sylvant OR infliximab OR Remicade OR Renflexis OR Inflectra OR Avakine OR Remsima OR Avsola OR Flixabi OR Renflexis OR "infliximab-dyyb" OR adalimumab OR Amjevita OR Cyltezo OR Humira OR Trudexa OR etanercept OR Enbrel OR Elrezi OR golimumab OR Simponi OR certolizumab OR Cimzia OR rituximab OR Mabthera OR Rituxan OR belimumab OR LymphoStat OR Benlysta OR anakinra OR Antril OR Kineret OR canakinumab OR Ilaris OR abatacept OR Belatacept OR Orencia OR Nulojix OR ustekinumab OR Stelara OR secukinumab OR Cosentyx OR ixekizumab OR Taltz OR guselkumab OR Tremfya OR tildrakizumab OR Ilumya OR Risankizumab OR skyrizi OR vedolizumab OR Entyvio OR omalizumab OR Xolair OR mepolizumab OR Bosatria OR Nucala OR benralizumab OR Fasenra OR rapamycin OR Rapamune OR sirolimus OR everolimus OR Afinitor OR Zortress OR Certican OR temsirolimus OR Torisel OR alemtuzumab OR Campath OR Lemtrada OR mitoxantrone OR Novantrone OR ocrelizumab OR Ocrevus OR natalizumab OR Tysabri OR "mycophenolic acid" OR mycophenolate OR Cellcept OR Myfortic OR azathioprine OR Imurel OR Imuran OR Immuran OR methotrexate OR Amethopterin OR Mexate OR hydroxychloroquine OR Oxychlorochin OR Oxychloroquine OR Hydroxychlorochin OR Plaquenil OR tacrolimus OR Prograf OR Prograft OR cyclosporine OR Neoral OR Sandimmun OR Sandimmune OR cyclosporin OR leflunomide OR Arava OR salazosulfapyridine OR Sulfazine OR Azulfidine OR Salicylazosulfapyridine OR Sulphasalazine OR Colo-Pleon OR Pleon OR sulfasalazine OR tofacitinib OR Xeljanz OR baricitinib OR Olumiant OR upadacitinib OR Rinvoq OR ruxolitinib OR Jakafi OR "intravenous immunoglobulin*" OR "IV immunoglobulin*" OR "IV gammaglobulin" OR "IVIG" OR Flebogamma OR Gamunex OR "Globulin-N" OR Intraglobin OR Gammagard OR Gamimune OR Gamimmune OR Privigen OR Sandoglobulin OR Venoglobulin OR Venimmune OR Iveegam OR Alphaglobin OR Endobulin OR Gammonativ OR upadacitinib OR Rinvoq OR ruxolitinib OR Jakafi OR plasmapheresis OR "therapeutic Immunoadsorption" OR "therapeutic plasma adsorption" OR apremilast OR Otezla OR cyclophosphamide OR Sendoxan OR Cytoxan OR Endoxan OR Neosar OR Procytox OR "thymocyte antibody" OR "anti-thymocyte globulin*" OR "antithymocyte globulin*" OR "anti-lymphocyte serum" OR "antilymphocyte serum" OR "antilymphocyte globulin*" OR "anti-lymphocyte globulin*" OR "anti-lymphocyte immunoglobulin*" OR "antilymphocyte immunoglobulin*" OR Pressimmune OR ATGAM)  AND  TS=("case series" OR "clinical stud*" OR "clinical trial*" OR retrospective* OR prospective* OR observational OR "comparative stud*" OR "follow up" OR longitudinal* OR multicenter OR cohort OR cohorts OR randomised OR randomized OR "record review*")  AND LA=(English)  NOT  (DT=(letter) OR DT=(Editorial Material) OR DT=(Meeting Abstract) OR (DT=(Review) NOT (DT=(Record Review) OR TS=("case series" OR "record review" OR “records review”))) OR TI=(“case report” OR “reported case”)) |
| **Cochrane Central Register of Controlled Trials** (Wiley)  537 references retrieved as of January 5, 2023. | Title Abstract Keyword: (ipilimumab OR yervoy OR "mdx-010" OR mdx010 OR "mdx-ctla-4" OR "mdx-ctla4" OR pembrolizumab OR keytruda OR lambrolizumab OR "sch-900475" OR sch900475 OR "mk-3475" OR mk3475 OR nivolumab OR opdivo OR "bms-936558" OR bms936558 OR "mdx-1106" OR mdx1106 OR "ono-4538" OR ono4538 OR atezolizumab OR "mdpl-3280a" OR mdpl3280a OR "rg-7446" OR rg7446 OR tecentriq OR avelumab OR bavencio OR "msb-0010718c" OR msb0010718c OR cemiplimab OR libtayo OR "regn-2810" OR regn2810 OR durvalumab OR imfinzi OR "medi-4736" OR medi4736 OR dostarlimab OR jemperli OR "gsk-4057190" OR gsk4057190 OR "tsr-042" OR tsr042 OR camrelizumab OR airuika OR "shr-1210" OR shr1210 OR ticilimumab OR tremelimumab OR "cp-675" OR cp675* OR checkpoint NEXT inhibit* OR checkpoint NEXT block* OR checkpoint NEXT antagonist* OR pd-l1 NEXT inhibitor* OR pdl1 NEXT inhibitor* OR "programmed death ligand 1" NEXT inhibitor* OR "programmed cell death ligand 1" NEXT inhibitor* OR pd-l1 NEXT antibod* OR pdl1 NEXT antibod* OR "programmed death ligand 1" NEXT antibod* OR "programmed cell death ligand 1" NEXT antibod* OR "anti pd l1" OR "anti-pdl1" OR "programmed death ligand 1" NEXT antagonist* OR "programmed cell death ligand 1" NEXT antagonist* OR "pd-l1" NEXT antagonist* OR pdl1 NEXT antagonist* OR "programmed death ligand 1" NEXT block* OR "programmed cell death ligand 1" NEXT block* OR "pd-l1" NEXT block* OR pdl1 NEXT block* OR "pd-1" NEXT inhibitor* OR pd1 NEXT inhibitor* OR "programmed cell death protein 1" NEXT inhibitor* OR "pd-1" NEXT antibod* OR pd1 NEXT antibod* OR "programmed cell death protein 1" NEXT antibod* OR "pd 1 pd l1" NEXT block* OR "anti-pd-1" OR "anti-pd1" OR "anti-programmed death" OR "antiprogrammed death" OR "pd-1" NEXT antagonist* OR pd1 NEXT antagonist* OR "programmed cell death protein 1" NEXT antagonist* OR "pd-1 receptor" NEXT block* OR "pd1 receptor" NEXT block* OR "pd-1" NEXT block* OR pd1 NEXT block* OR "programmed cell death 1 receptor" NEXT block* OR "pd-1 receptor" NEXT antagonist* OR "pd1 receptor" NEXT antagonist* OR "programmed cell death 1 receptor" NEXT antagonist* OR "pd-1 receptor" NEXT antibod* OR "pd1 receptor" NEXT antibod* OR "programmed cell death 1 receptor" NEXT antibod* OR "ctla-4" NEXT inhibitor* OR ctla4 NEXT inhibitor* OR "cytotoxic t-lymphocyte-associated protein 4" NEXT inhibitor* OR "cytotoxic t lymphocyte associated antigen 4" NEXT inhibitor* OR "cytotoxic t-lymphocyte antigen 4" NEXT inhibitor* OR "ctla-4" NEXT antibod* OR ctla4 NEXT antibod* OR "cytotoxic t-lymphocyte-associated protein 4" NEXT antibod* OR "cytotoxic t lymphocyte-associated antigen 4" NEXT antibod* OR "cytotoxic t lymphocyte antigen 4" NEXT antibod* OR "anti-ctla-4" OR "anti-ctla4" OR "anti-cytotoxic t-lymphocyte-associated protein 4" OR "anti-cytotoxic t lymphocyte associated" NEXT antigen* OR "anti-cytotoxic t-lymphocyte antigen 4" OR ctla-4 NEXT antagonist* OR ctla4 NEXT antagonist* OR "ctla-4 antigen" NEXT antagonist* OR "cytotoxic t-lymphocyte-associated protein 4" NEXT antagonist* OR "cytotoxic t lymphocyte–associated antigen 4" NEXT antagonist* OR "cytotoxic t lymphocyte antigen 4" NEXT antagonist* OR "ctla-4 antigen" NEXT block* OR "cytotoxic t-lymphocyte-associated protein 4" NEXT block* OR "cytotoxic t lymphocyte–associated antigen 4" NEXT block* OR "cytotoxic t lymphocyte antigen 4" NEXT block* OR "ctla-4" NEXT block* OR ctla4 NEXT block*)  AND  Title Abstract Keyword: (tocilizumab OR Actemra OR Roactemra OR Atlizumab OR sarilumab OR Kevzara OR siltuximab OR Sylvant OR infliximab OR Remicade OR Renflexis OR Inflectra OR Avakine OR Remsima OR Avsola OR Flixabi OR Renflexis OR infliximab-dyyb OR adalimumab OR Amjevita OR Cyltezo OR Humira OR Trudexa OR etanercept OR Enbrel OR Elrezi OR golimumab OR Simponi OR certolizumab OR Cimzia OR rituximab OR Mabthera OR Rituxan OR belimumab OR LymphoStat OR Benlysta OR anakinra OR Antril OR Kineret OR canakinumab OR Ilaris OR abatacept OR Belatacept OR Orencia OR Nulojix OR ustekinumab OR Stelara OR secukinumab OR Cosentyx OR ixekizumab OR Taltz OR guselkumab OR Tremfya OR tildrakizumab OR Ilumya OR Risankizumab OR skyrizi OR vedolizumab OR Entyvio OR omalizumab OR Xolair OR mepolizumab OR Bosatria OR Nucala OR benralizumab OR Fasenra OR rapamycin OR Rapamune OR sirolimus OR everolimus OR Afinitor OR Zortress OR Certican OR temsirolimus OR Torisel OR alemtuzumab OR Campath OR Lemtrada OR mitoxantrone OR Novantrone OR ocrelizumab OR Ocrevus OR natalizumab OR Tysabri OR "mycophenolic acid" OR mycophenolate OR Cellcept OR Myfortic OR azathioprine OR Imurel OR Imuran OR Immuran OR methotrexate OR Amethopterin OR Mexate OR hydroxychloroquine OR Oxychlorochin OR Oxychloroquine OR Hydroxychlorochin OR Plaquenil OR tacrolimus OR Prograf OR Prograft OR cyclosporine OR Neoral OR Sandimmun OR Sandimmune OR cyclosporin OR leflunomide OR Arava OR salazosulfapyridine OR Sulfazine OR Azulfidine OR Salicylazosulfapyridine OR Sulphasalazine OR Colo-Pleon OR Pleon OR sulfasalazine OR tofacitinib OR Xeljanz OR baricitinib OR Olumiant OR upadacitinib OR Rinvoq OR ruxolitinib OR Jakafi OR intravenous NEXT immunoglobulin* OR IV NEXT immunoglobulin* OR IV NEXT gammaglobulin* OR IVIG OR Flebogamma OR Gamunex OR Globulin-N OR Intraglobin OR Gammagard OR Gamimune OR Gamimmune OR Privigen OR Sandoglobulin OR Venoglobulin OR Venimmune OR Iveegam OR Alphaglobin OR Endobulin OR Gammonativ OR upadacitinib OR Rinvoq OR ruxolitinib OR Jakafi OR plasmapheresis OR "therapeutic Immunoadsorption" OR "therapeutic plasma adsorption" OR apremilast OR Otezla OR cyclophosphamide OR Sendoxan OR Cytoxan OR Endoxan OR Neosar OR Procytox OR "thymocyte antibody" OR "anti-thymocyte" NEXT globulin* OR antithymocyte NEXT globulin* OR "anti-lymphocyte serum" OR "antilymphocyte serum" OR antilymphocyte NEXT globulin* OR "anti-lymphocyte" NEXT globulin* OR "anti-lymphocyte" NEXT immunoglobulin* OR antilymphocyte NEXT immunoglobulin* OR Pressimmune OR ATGAM) |

Note: Subsequent to protocol registration, the decision was made to exclude case reports and conference abstracts.

Supplement 2

**Supplement 2a: Studies with sufficient individual level data analysis**

|  | Lead author, year | Tumor type | SSIA(s) | irAE | Tumor outcomes |
| --- | --- | --- | --- | --- | --- |
|  | TNFi |  |  |  |  |
| 1 | Johncilla, 2020 | Melanoma | Infliximab | Gastritis | OS, PFS |
| 2 | Lesage, 2019 | Melanoma | Infliximab | Colitis | OS, OSIRAE |
| 3 | Lin, 2021 | Mixed | Infliximab | Nephritis | PFS, OS, RECIST, OSIRAE |
| 4 | Miyahara, 2020 | NSCLC | Infliximab | Colitis | PFS, OS, RECIST, OSIRAE |
| 5 | Sagiv, 2018 | Melanoma | Infliximab | Colitis | PFS, OS, RECIST |
|  | IVIG |  |  |  |  |
| 6 | Galmiche, 2019 | Melanoma | IVIG | Encephalitis | PFS, OS, RECIST, OSIRAE |
| 7 | Mohn, 2019 | Melanoma | IVIG | Neurologic | PFS, OS, RECIST, OSIRAE |
| 8 | Nakagomi, 2022 | Mixed | IVIG | Myositis/Myocarditis | OS, PFS, RECIST |
|  | Methotrexate |  |  |  |  |
| 9 | Lidar, 2018 | Mixed | Methotrexate | Mixed | PFS, OS, RECIST |
| 10 | O'Reilly, 2020 | Melanoma | Methotrexate | Mixed | RECIST |
|  | Mycophenolate |  |  |  |  |
| 11 | Izumi, 2019 | NSCLC | Mycophenolate | Cholangitis | OS, RECIST |
|  | Mixed |  |  |  |  |
| 12 | Camard, 2022 | Mixed | Mixed | Pneumonitis | OS |
| 13 | Campochiaro, 2021 | Lung | Mixed | Mixed | PFS, OS, RECIST |
| 14 | Cortazar, 2020 | Mixed | Mixed | Nephritis | OS |
| 15 | De La Fuente, 2022 | Mixed | Mixed | Arthritis | PFS, OS, RECIST, OSIRAE |
| 16 | Kim, 2019 | Melanoma | IVIG, infliximab | Mixed | PFS, OS, RECIST, OSIRAE |
| 17 | Larkin, 2017 | Melanoma | IVIG and rituximab | Encephalitis | PFS, OS, RECIST, OSIRAE |
| 18 | Luo, 2021 | NSCLC | Mixed | Mixed | PFS, OS, RECIST, OSIRAE |
| 19 | Mamlouk, 2020 | Mixed | Rituximab, PLEX | Renal vasculitis | OS, OSIRAE |
| 20 | Mitchell, 2018 | Mixed | Mixed | Mixed | PFS |
| 21 | Segui, 2022 | Mixed | Mixed | Mixed | PFS, OS |
| 22 | Taliansky, 2021 | Mixed | Cyclophosphamide, PLEX | Neurologic | PFS, OS |

**Supplement 2b: Studies for group level data analysis**

|  | Lead author, year | Tumor type | SSIA(s) | irAE | Tumor outcomes |
| --- | --- | --- | --- | --- | --- |
| 1 | Abu-Sbeih, 2019 | Mix | TNFi (Infliximab +/- Vedo vs. Vedo) | Colitis | Estimate OS from KM |
| 2 | Alexander, 2021 | Mix | TNFi (infliximab) +/- other SSIAs | Colitis | 12mo PFS, 12mo ORR |
| 3 | Arriola, 2015 | Melanoma | TNFi (Infliximab) | Mixed | Estimate OS from KM |
| 4 | Bass, 2023 | Melanoma | TNFi (Any) | Arthritis | 12mo PFS |
|  | Bass, 2023 | Melanoma | Mix of SSIAs | Arthritis | 6mo/12mo/24mo OS, 12mo PFS |
| 5 | Burdett, 2020 | Mix | Mix of SSIAs | Mixed | mOS, Estimate OS, ORR, RECIST |
| 6 | Chan, 2020 | Mix | Mix of SSIAs (cDMARDs) | Mixed | ORR, RECIST |
| 7 | Cheung, 2019 | Mix | TNFi (Infliximab) + MMF / tacrolimus | Hepatitis | Estimate OS from KM |
| 8 | Cheung, 2020 | Mix | TNFi (Infliximab) | Colitis | Estimate OS from KM |
| 9 | Dahl, 2022 | Mix | TNFi (Infliximab) +/- vedolizumab | Colitis | mOS, Estimate OS, 12mo OS, 12mo PFS |
| 10 | Dearden, 2021 | Melanoma | Mix of SSIAs | Mixed | mOS, 12mo OS, mPFS, 12mo PFS, ORR |
| 11 | Dimitriou, 2021 | Melanoma | Mix of SSIAs (Inflix or Toci) | Mixed | Estimate OS, Estimate PFS |
| 12 | Hughes, 2019 | Melanoma | Mix of SSIAs | Colitis | mOS, Estimate OS, 20mo OS,  mPFS, Estimate PFS, 20mo PFS |
| 13 | Johnson, 2018 | Melanoma | TNFi (Infliximab) | Colitis | mOS, Estimate OS, 20mo OS,  mPFS, Estimate PFS, 20mo PFS |
| 14 | Lesage, 2019 | Melanoma | TNFi (Any) | Colitis | mOS, 12mo OS, mPFS, 6mo/12mo ORR |
| 15 | Mooradian, 2020 | Melanoma | TNFi (Infliximab) | Colitis | Estimate OS, 12mo OS, 24mo OS |
| 16 | Nahar, 2020 | Melanoma | TNFi (Inflix sensitive, Infliximab refractory) | Colitis | Estimate OS, 20mo OS, Estimate PFS, 20mo PFS, ORR |
| 17 | Stroud, 2017 | Lung NOS | Toci | Mixed | *aHR OS,* mOS, Estimate OS |
| 18 | van Not, 2022 | Melanoma | Mix of SSIAs TNFi (infliximab) | Mixed | *aHR OS,* mOS, 6/12/24mo OS,  *aHR PFS,* mPFS, 6/12/24mo PFS |
|  | van Not, 2022 | Melanoma | TNFi (Infliximab) | Mixed | *aHR OS,* mOS, 6/12/24mo OS,  *aHR PFS,* mPFS, 6/12/24mo PFS |
| 19 | Verheijden, 2020 | Melanoma | TNFi (Any) | Mixed | *aHR OS,* mOS, Estimate OS, 20mo OS |
| 20 | Wang, 2018 | Mix | TNFi (Infliximab) | Colitis | Estimate OS |
| 21 | Zhang, 2021 | Melanoma | TNFi (Any) | Colitis | Estimate OS, 12mo OS, RECIST |
| 22 | Zou, 2021 | Mix | TNFi (Inf only. Other: Vedo, Inf+vedo) | Colitis | *aHR OS,* Estimate OS *aHR PFS* |

Estimate OS: signifies that OS details would have to be estimated+calculated from the KM curve in the manuscript; Estimate PFS: signifies that PFS details would have to be estimated+calculated from the KM curve in the manuscript.

aHR=adjusted hazard ratio; mOS= median OS; mPFS=median PFS; ORR=objective response rate.

Supplement 3

**Quality Assessment**

|  | Authors | Assessment |
| --- | --- | --- |
|  | Cohort Studies |  |
| 1 | Abu-Sbeih, 2019 | Poor |
| 2 | Alexander, 2021 | Poor |
| 3 | Arriola, 2015 | Good |
| 4 | Bass, 2023 | Poor |
| 5 | Camard, 2022 | Good |
| 6 | Cortazar, 2020 | Good |
| 7 | Chan, 2020 | Poor |
| 8 | Cheung, 2019 | Good |
| 9 | Cheung, 2020 | Good |
| 10 | Dahl, 2022 | Good |
| 11 | De La Fuente F, 2022 | Poor |
| 12 | Dearden, 2021 | Poor |
| 13 | Dimitriou, 2021 | Good |
| 14 | Galmiche, 2019 | Poor |
| 15 | Hughes, 2019 | Good |
| 16 | Johncilla, 2020 | Poor |
| 17 | Johnson, 2018 | Good |
| 18 | Lesage, 2019 | Poor |
| 19 | Lidar, 2018 | Poor |
| 20 | Lin, 2021 | Poor |
| 21 | Luo, 2021 | Poor |
| 22 | Mamlouk, 2020 | Poor |
| 23 | Mooradian, 2020 | Poor |
| 24 | Nahar, 2020 | Poor |
| 25 | O'Reilly, 2020 | Good |
| 26 | Stroud, 2017 | Poor |
| 27 | Taliansky, 2021 | Poor |
| 28 | van Not, 2022 | Good |
| 29 | Verheijden, 2020 | Good |
| 30 | Wang, 2018 | Good |
| 31 | Zou, 2021 | Good |
|  | Case Series |  |
| 32 | Burdett, 2020 | Good |
| 33 | Campochiaro, 2021 | Fair |
| 34 | Izumi, 2019 | Poor |
| 35 | Kim, 2019 | Fair |
| 36 | Larkin, 2017 | Good |
| 37 | Mitchell, 2018 | Fair |
| 38 | Miyahara, 2020 | Fair |
| 39 | Mohn, 2019 | Fair |
| 40 | Nakagomi, 2022 | Fair |
| 41 | Sagiv, 2018 | Poor |
| 42 | Segui, 2022 | Fair |
| 43 | Zhang, 2021 | Poor |

Supplement 4

**Individual-level missing data, n(%)**

|  | Overall  (n=236) | No SSIA  (n= 62) | SSIA  (n= 174) | p |
| --- | --- | --- | --- | --- |
| Demographics |  |  |  |  |
| Age | 116 (49.2) | 12 (19.4) | 104 (59.8) | <0.001 |
| Sex | 116 (49.2) | 12 (19.4) | 104 (59.8) | <0.001 |
| Cancer Details |  |  |  |  |
| Malignancy type | 17 (7.2) | 0 (0.0) | 17 (9.8) | <0.001 |
| ICI type | 19 (8.1) | 0 (0.0) | 19 (10.9) | 0.012 |
| ICI Duration | 132 (55.9) | 30 (48.4) | 102 (58.6) | 0.213 |
| ICI Discontinuation | 101 (42.8) | 12 (19.4) | 89 (51.1) | <0.001 |
| Additional therapy received (y/n) | 123 (52.1) | 35 (56.5) | 88 (50.6) | 0.022 |
| RECIST | 104 (44.1) | 39 (62.9) | 65 (37.4) | 0.027 |
| irAE Details |  |  |  |  |
| Time to irAE | 37 (15.7) | 21 (33.9) | 16 (9.2) | <0.001 |
| Time to Steroids | 139 (58.9) | 47 (75.8) | 92 (52.9) | 0.003 |
| Time to SSIA |  |  | 100 (57.5) |  |
| Duration SSIA |  |  | 55 (31.6) |  |
| irAE Outcome | 108 (45.8) | 13 (21.0) | 95 (54.6) | <0.001 |
| Survival |  |  |  |  |
| PFS | 35 (14.8) | 9 (14.5) | 26 (14.9) | 0.781 |
| OS | 23 (9.7) | 14 (22.6) | 9 (5.2) | <0.001 |

Supplement 5

**OS and PFS after SSIA vs. CS monotherapy for melanoma *without adjusting for immortal time bias***

1. **OS for SSIA vs. CS monotherapy for melanoma, *without adjusting for immortal time bias***


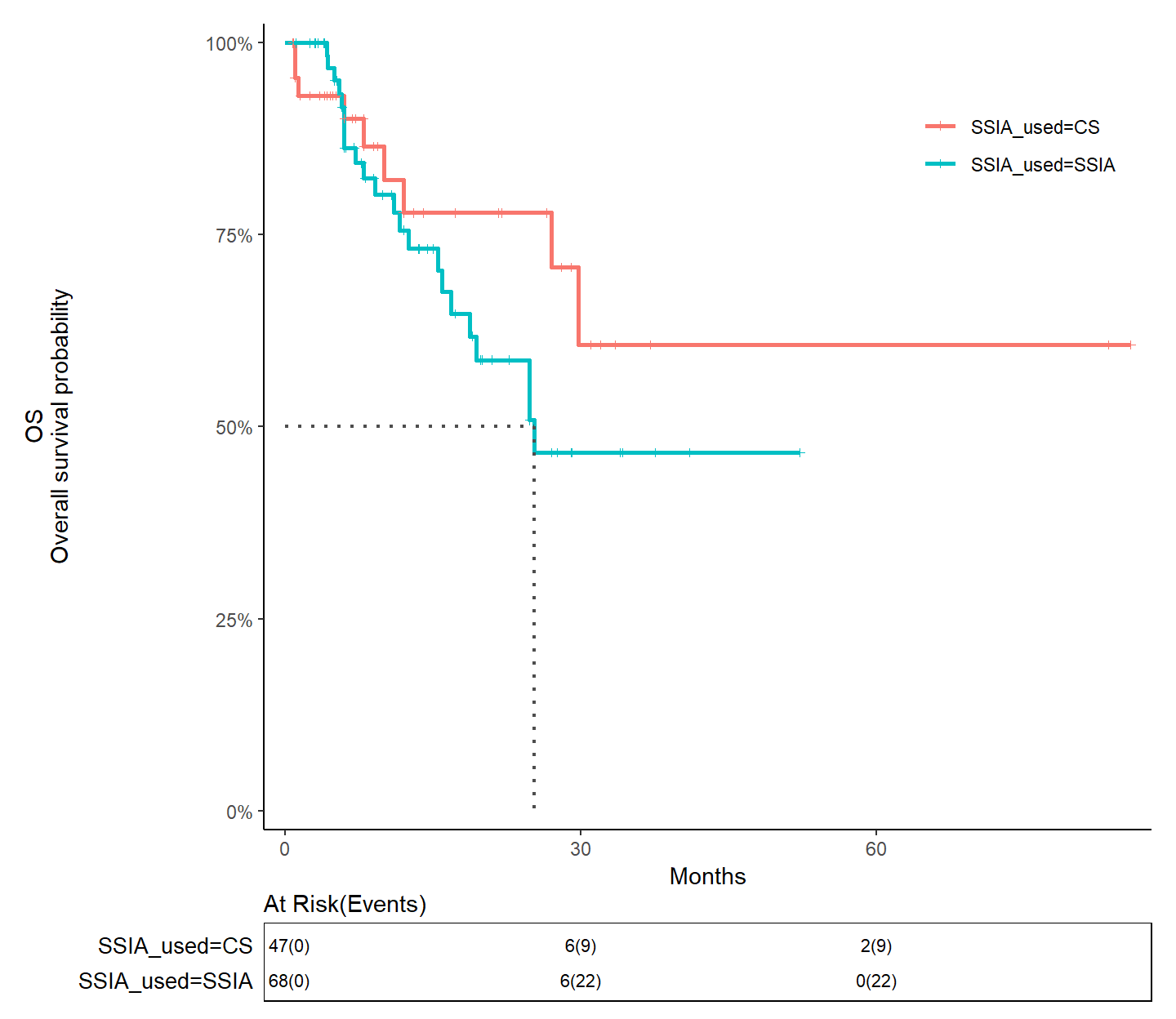


1. **PFS after SSIA vs. CS monotherapy for melanoma, *without adjusting for immortal time bias***


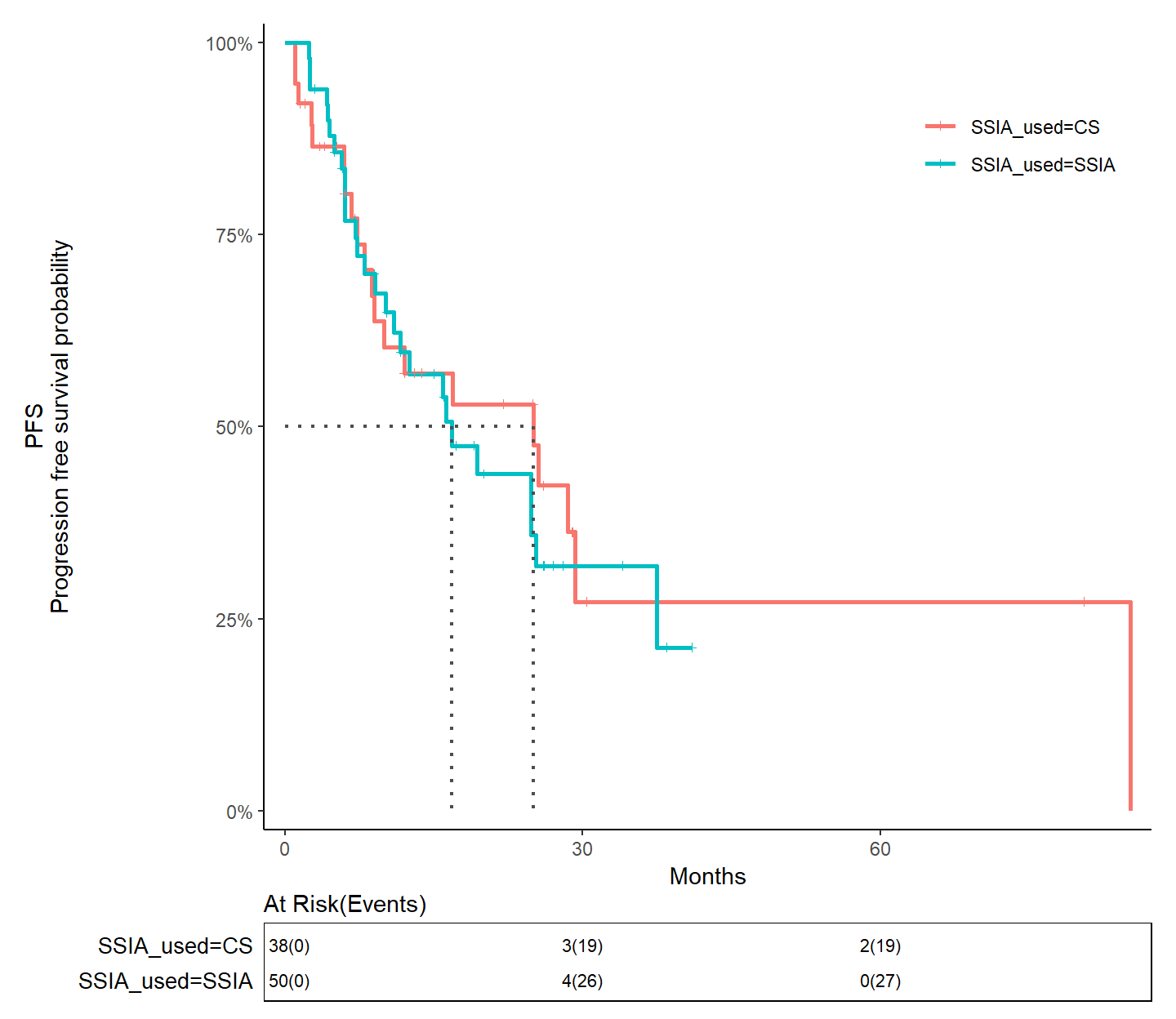


Supplement 6

Group level data for any SSIA

| Article | Authors | Journal | Year | Median OS SSIA | Median OS NO SSIA | aHR OS | Median PFS SSIA | Median PFS NO SSIA | aHR PFS |
| --- | --- | --- | --- | --- | --- | --- | --- | --- | --- |
| Clinical outcomes of patients with corticosteroid refractory immune checkpoint inhibitor-induced enterocolitis treated with infliximab. | Alexander, et al. | J Immunother Cancer | 2021 | - | - | - | - | - | - |
| Cancer outcomes in patients requiring immunosuppression in addition to corticosteroids for immune-related adverse events after immune checkpoint inhibitor therapy. | Burdett, et al. | Asia Pac J Clin Oncol | 2020 | 9.4 months | NA | NA | - | - | - |
| Higher Checkpoint Inhibitor Arthritis Disease Activity may be Associated With Cancer Progression: Results From an Observational Registry | Chan, et al. | ACR Open Rheumatology | 2020 | - | - | - | - | - | - |
| Incidence and Clinical Impact of Anti-TNF Treatment of Severe Immune Checkpoint Inhibitor-induced Colitis in Advanced Melanoma: The Mecolit Survey. | Lesage, et al. | J Immunother | 2019 | 12 months | - | - | 3 months | - | - |
| Calcineurin inhibitors in steroid and anti-TNF-alpha refractory immune checkpoint inhibitor colitis. | Zhang, et al. | JGH Open | 2021 | - | NA | NA | - | NA | NA |
| Comparative safety and effectiveness of il6r inhibitors, tnf inhibitors and methotrexate for the treatment of immune checkpoint inhibitorassociated arthritis | Bass, et al. ANY CANCER | Ann Rheum Dis | 2023 | Not reached | - | - | not reached | - | - |
| Safety and efficacy of infliximab and corticosteroid therapy in checkpoint inhibitor-induced colitis. | Dahl, et al. | Aliment Pharmacol Ther. | 2022 | 27.87 mo (95% CI 620-1232 days) | 13 mo (341-462d) | - | - | - | - |
| Efficacy and safety of vedolizumab and infliximab treatment for immune- mediated diarrhea and colitis in patients with cancer: a two-center observational study | Zou, et al. | J Immunother | 2021 | 29.15 mo E | Not reached | 2.04 (CI 1.15-3.62), p=0.014 | - | - | 5.24 (CI 2.33-11.77) OR p<001 |
| Early introduction of selective immunosuppressive therapy associated with favorable clinical outcomes in patients with immune checkpoint inhibitor-induced colitis. | Abu-Sbeih, et al. | J Immunother Cancer | 2019 | Not reached | Not reached | - (p=0.151) | - | - | - |
| Infliximab for IPILIMUMAB-related colitis-letter | Arriola, et al. | Clinical Cancer Research | 2015 | Not reached | 7 mo (95%CI, 3 – 11) | - p=0.2 | - | - | - |
| Immunotherapy-related hepatitis: real-world experience from a tertiary centre. | Cheung, et al. | Frontline Gastroenterol | 2019 | Not reached | 15.3 mo E | - (p=0.08) | - | - | - |
| Immune checkpoint inhibitor-related colitis assessment and prognosis: can IBD scoring point the way? | Cheung, et al. | Br J cancer | 2020 | Not reached | E from KM supp 6d | - (p=0.4) | - | - | - |
| Hyperacute toxicity with combination ipilimumab and anti-PD1 immunotherapy | Dearden, et al. | EUROPEAN JOURNAL OF CANCER | 2021 | 2.9 months | - | - | 2.9 months | 7.1 months | - (p=.0006) |
| Frequency, Treatment and Outcome of Immune-Related Toxicities in Patients with Immune-Checkpoint Inhibitors for Advanced Melanoma: Results from an Institutional Database Analysis. | Dimitriou, et al. | Cancers (Basel) | 2021 | H2E fig 13 | H2E fig 14 | H2E fig 15 | H2E fig 20 | H2E fig 21 | - |
| Colitis after checkpoint blockade: A retrospective cohort study of melanoma patients requiring admission for symptom control | Hughes, et al. | Cancer Medicine | 2019 | 54.6mo (13.7-NE) | 35.6mo (12.2-NE) | - (p=0.4) | 30.6mo (6.5-NE) | 10.8mo (4.8-NE) | - (P = 0.367 log‐ rank, 0.174 Wilcoxon)" |
| Infliximab associated with faster symptom resolution compared to corticosteroids alone for management of immune related enterocolitis | Johnson, et al. | J Immunother Cancer | 2018 | Not reached | Not reached | - | 9 mo (95%CI 5.6- Not reached) | 12.5 mo (95%CI 5.8-not reached) | - |
| Mucosal inflammation predicts response to systemic steroids in immune checkpoint inhibitor colitis. | Mooradian, et al. | J Immunother Cancer | 2020 | E KM 5b | E KM 5b | - | - | - | - |
| Clinicopathological characteristics and management of colitis with anti-PD1 immunotherapy alone or in combination with ipilimumab. | Nahar, et al. | J Immunother Cancer | 2020 | Not reached | Not reached | - |  |  |  |
| Tocilizumab for the management of immune mediated adverse events secondary to PD-1 blockade | Stroud, et al. | Journal of Clinical Oncology | 2017 | 6.1 months | 8.7 months | 1.28 (95CI 0.74-2.22), p=0.37 | - | - | - |
| Association of Immune-Related Adverse Event Management With Survival in Patients With Advanced Melanoma | van Not, et al.ANY SSIA | JAMA oncology | 2022 | 22.5 mo (36.5-not reached) | 46.1 mo (95%CI 39-Not reached) | 1.54 (CI 1.03-2.30), p=0.04 | 5.4 mo (95% CI 4.5-12.4) | 11.3 mo (95% CI 9.6-19.5) | 1.40 (95% CI 1.00-1.97), p=0.05 |
| Association of Anti-TNF with Decreased Survival in Steroid Refractory Ipilimumab and Anti-PD1-Treated Patients in the Dutch Melanoma Treatment Registry - PubMed (nih.gov) | Verheijden, et al. | Clinical Cancer Research | 2020 | 17 mo | 27 mo | 1.61 (CI 1.03-2.51) | - | - | - |
| Immune-checkpoint inhibitor-induced diarrhea and colitis in patients with advanced malignancies: Retrospective review at MD Anderson | Wang, et al. | J Immunother Cancer | 2018 | E from KM 1d | Not reached | - (p=0.768) | - | - | - |

H2E: hard to estimate

E: Estimated from KM curve

NA: Not available

Supplement 7

1. **OS and PFS for TNFi versus Steroids only and TNFi versus Other SSIAs for melanoma *without adjusting for immortal time bias***


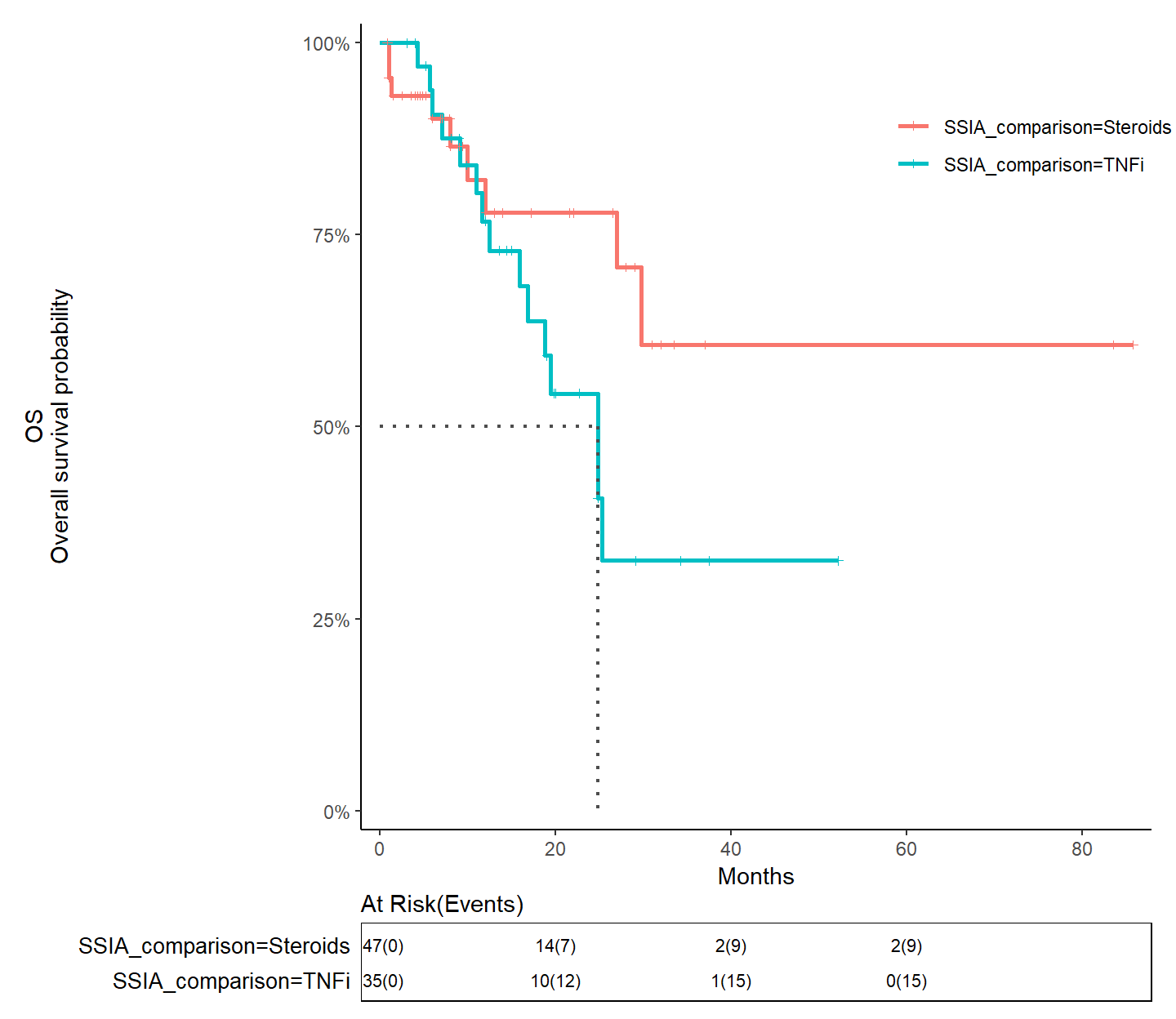


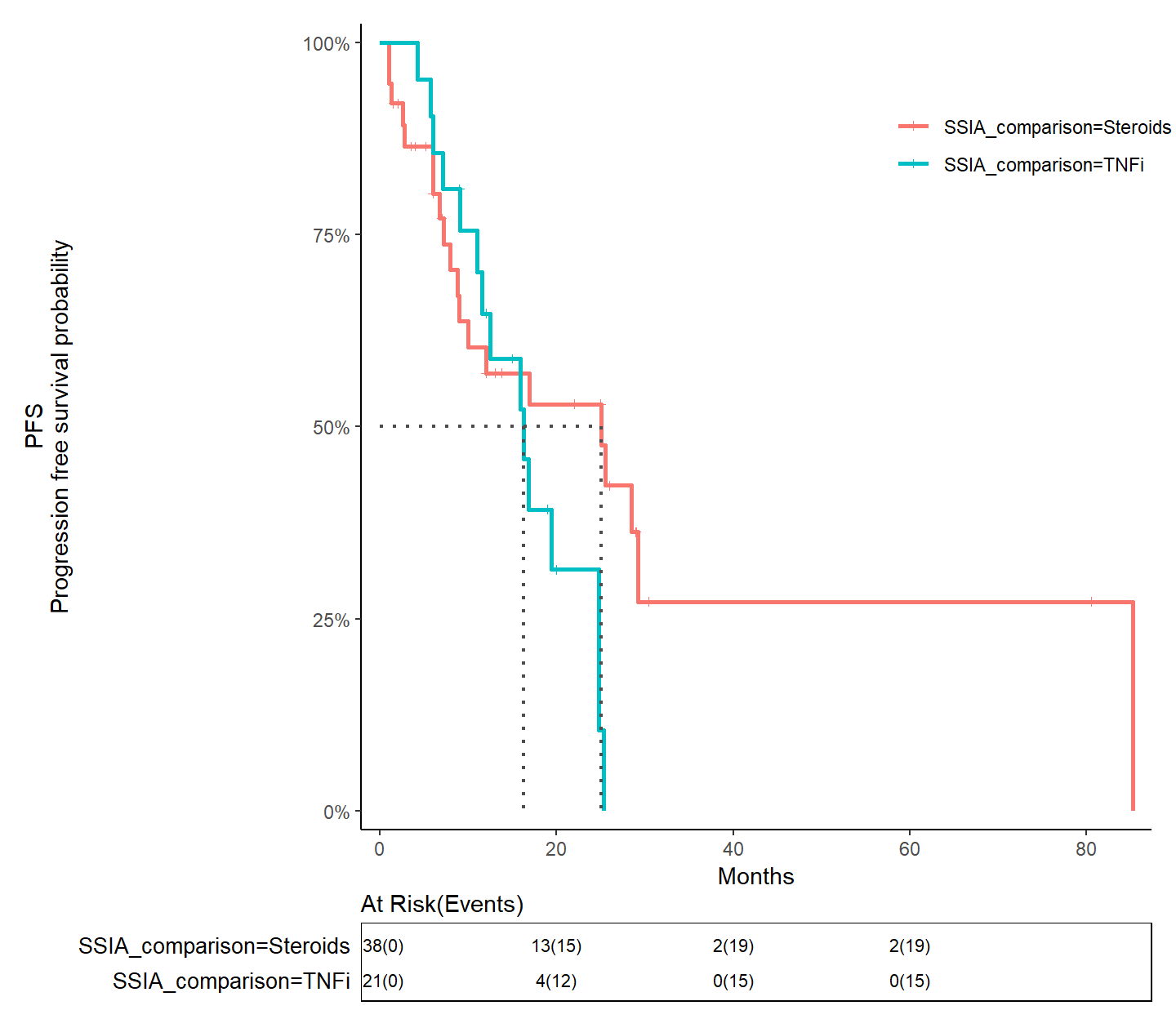


1. **OS and PFS for TNFi versus Other SSIAs for melanoma *without adjusting for immortal time bias***


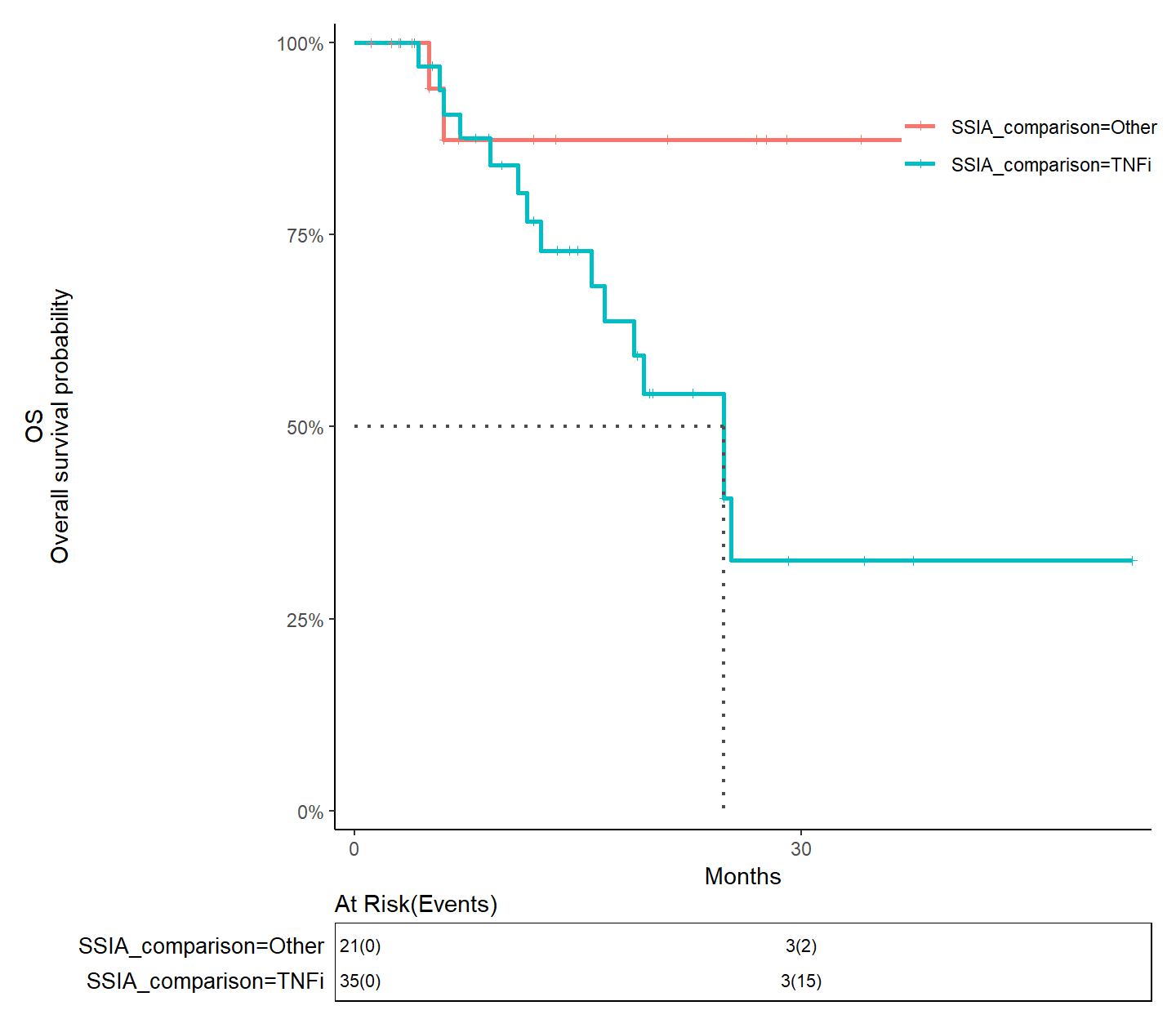


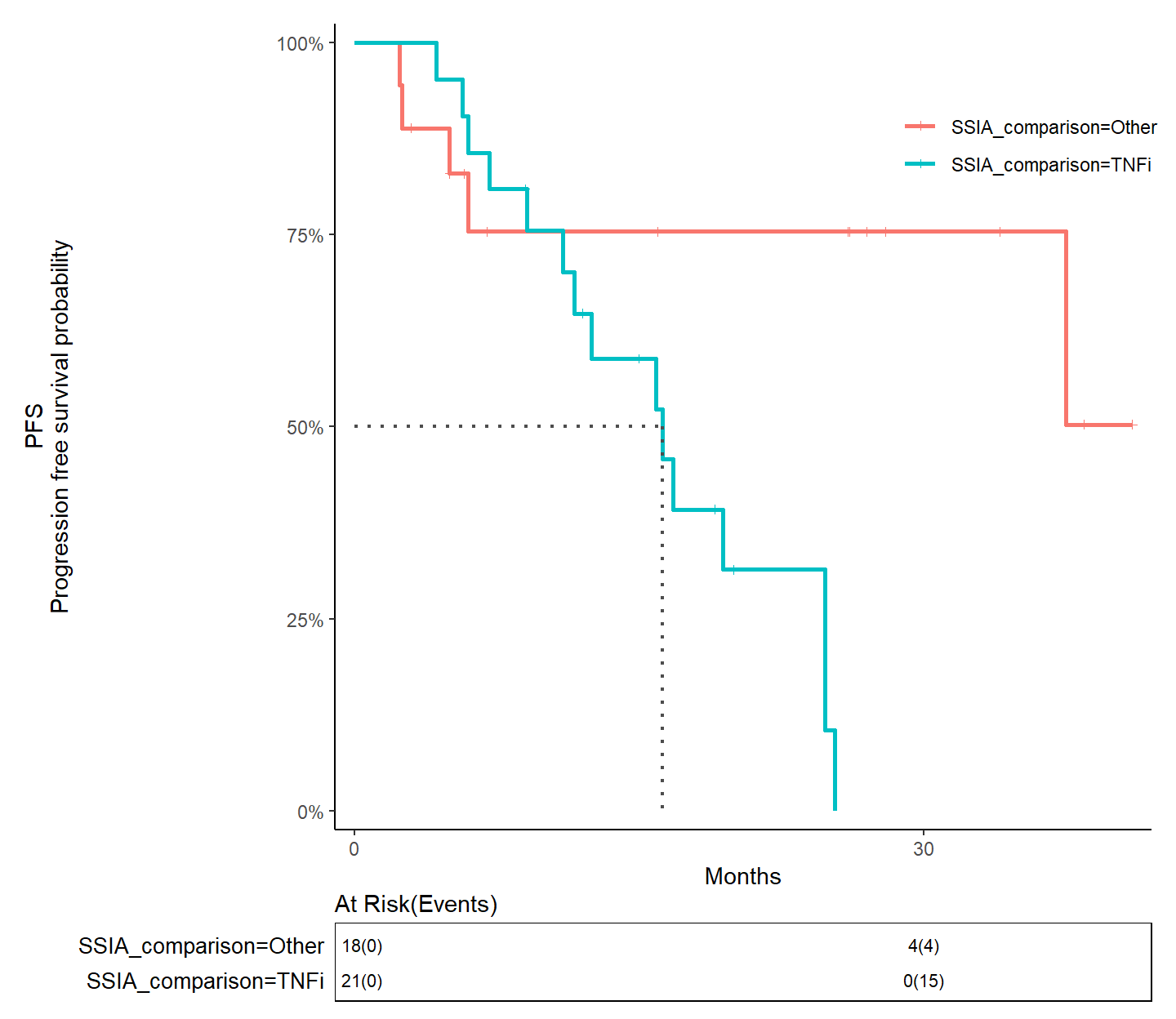


Supplement 8

**Group level data for TNFi**

| Article | Authors | Journal | Year | Median OS SSIA | Median OS NO SSIA | aHR OS | Median PFS SSIA | Median PFS NO SSIA | aHR Median PFS |
| --- | --- | --- | --- | --- | --- | --- | --- | --- | --- |
| Calcineurin inhibitors in steroid and anti-TNF-alpha refractory immune checkpoint inhibitor colitis. | Zhang, et al. | JGH Open | 2021 | - | NA | NA | - | NA | NAuu |
| Incidence and Clinical Impact of Anti-TNF Treatment of Severe Immune Checkpoint Inhibitor-induced Colitis in Advanced Melanoma: The Mecolit Survey. | Lesage, et al. | J Immunother | 2019 | 12 months | - | - | 3 months | - | - |
| Clinical outcomes of patients with corticosteroid refractory immune checkpoint inhibitor-induced enterocolitis treated with infliximab. | Alexander, et al. | J Immunother Cancer | 2021 | - | - | - | - | - | - |
| Comparative safety and effectiveness of il6r inhibitors, tnf inhibitors and methotrexate for the treatment of immune checkpoint inhibitorassociated arthritis | Bass, et al. MELANOMA ONLY | Ann Rheum Dis | 2023 | - | - | - | not reached | - | - |
| Safety and efficacy of infliximab and corticosteroid therapy in checkpoint inhibitor-induced colitis. | Dahl et al. | Aliment Pharmacol Ther. | 2022 | 27.87 mo (95% CI 620-1232 days) | 13 mo (341-462d) | - | - | - | - |
| Efficacy and safety of vedolizumab and infliximab treatment for immune- mediated diarrhea and colitis in patients with cancer: a two-center observational study | Zou, et al. | J Immunother | 2021 | 29.15 mo E | Not reached | 2.04 (CI 1.15-3.62), p=0.014 | - | - | 5.24 (CI 2.33-11.77) OR p<001 |
| Early introduction of selective immunosuppressive therapy associated with favorable clinical outcomes in patients with immune checkpoint inhibitor-induced colitis. | Abu-Sbeih, et al. | J Immunother Cancer | 2019 | Not reached | Not reached | - (p=0.151) | - | - | - |
| Association of Anti-TNF with Decreased Survival in Steroid Refractory Ipilimumab and Anti-PD1-Treated Patients in the Dutch Melanoma Treatment Registry - PubMed (nih.gov) | Verheijden, et al. | Clinical Cancer Research | 2020 | 17 mo | 27 mo | 1.61 (CI 1.03-2.51) | - | - | - |
| Clinicopathological characteristics and management of colitis with anti-PD1 immunotherapy alone or in combination with ipilimumab. | Nahar, et al. | J Immunother Cancer | 2020 | Not reached | Not reached | - |  |  |  |
| Association of Immune-Related Adverse Event Management With Survival in Patients With Advanced Melanoma | van Not, et al. TNFi comparison only | JAMA oncology | 2022 | 31.7 mo (95% CI 15.7-not reached) | Not reached (95%CI 46.1-not reached) | 1.44 (CI 0.84-2.49), p=0.19 | 5.4 mo (95% CI 4.7-13.1) | 11.3 mo (95% CI 9.6-19.5) | 1.44 (95% CI 0.92-2.26), p=0.11 |
| Mucosal inflammation predicts response to systemic steroids in immune checkpoint inhibitor colitis. | Mooradian, et al. | J Immunother Cancer | 2020 | E KM 5b | E KM 5b | - | - | - | - |
| Infliximab associated with faster symptom resolution compared to corticosteroids alone for management of immune related enterocolitis | Johnson, et al. | J Immunother Cancer | 2018 | Not reached | Not reached | - | 9 mo (95%CI 5.6- Not reached) | 12.5 mo (95%CI 5.8-not reached) | - |
| Infliximab for IPILIMUMAB-related colitis-letter | Arriola, et al. | Clinical Cancer Research | 2015 | Not reached | 7 mo (95%CI, 3 – 11) | - p=0.2 | - | - | - |
| Immune-checkpoint inhibitor-induced diarrhea and colitis in patients with advanced malignancies: Retrospective review at MD Anderson | Wang, et al. | J Immunother Cancer | 2018 | E from KM 1d | Not reached | - (p=0.768) | - | - | - |
| Immune checkpoint inhibitor-related colitis assessment and prognosis: can IBD scoring point the way? | Cheung, et al. | Br J cancer | 2020 | Not reached | E from KM supp 6d | - (p=0.4) | - | - | - |
| Immunotherapy-related hepatitis: real-world experience from a tertiary centre. | Cheung, et al. | Frontline Gastroenterol | 2019 | Not reached | 15.3 mo E | - (p=0.08) | - | - | - |

Supplement 9

**Individual Level Data of Patients treated with IL6Ri**

| ID/Reference | Age | Sex | Malignancy_type | ICI_groups | ICI_duration (mo) | Primary_irAE_simp | Time_to_irAE | Second_SSIA | Time_irAE_to_SSIA | Duration_SSIA | Cancer_progression_after_SSIA | PFS | TTD | OS (from ICI start) |
| --- | --- | --- | --- | --- | --- | --- | --- | --- | --- | --- | --- | --- | --- | --- |
| Campochiaro1 | 69 | M | NSCLC | Monotherapy PD(L)1 or CTLA4 | 3 | Myocarditis | 3 | Mycophenolate | 8 | 10 | y | 21 | NA | 21 |
| Campochiaro5 | 75 | F | Other | Monotherapy PD(L)1 or CTLA4 | 1 | Vasculitis | 1 | NA | 3 | 7 | n | 12 | NA | 11 |
| DeLaFuente16 | NA | NA | NA | NA | 48 | Arthritis | 39 | NA | 4 | 2 | n | 48 | 46 | 48 |
| DeLaFuente18 | NA | NA | NA | NA | 0.25 | Arthritis | 0 | NA | 0.5 | 5.5 | y | 5 | 0.25 | 6 |
| DeLaFuente4 | NA | NA | NA | NA | 18 | Arthritis | 4 | NA | 12.5 | 8.5 | y | 20 | 12 | 23 |
| DeLaFuente5 | NA | NA | NA | NA | 6 | Arthritis | 0.75 | NA | 2.25 | 13.3 | n | 30 | 7.5 | 30 |
| Sequi17 | 79 | M | Melanoma | Monotherapy PD(L)1 or CTLA4 | NA | Myositis/Myocarditis | NA | IVIg | NA | NA | y | NA | NA | 6 |
